# Translational and Posttranslational Dynamics in a Model Peptidergic System

**DOI:** 10.1016/j.mcpro.2023.100544

**Published:** 2023-04-06

**Authors:** Soledad Bárez-López, André S. Mecawi, Natasha Bryan, Audrys G. Pauža, Victor J. Duque, Benjamin T. Gillard, David Murphy, Michael P. Greenwood

**Affiliations:** 1 Molecular Neuroendocrinology Research Group, Bristol Medical School: Translational Health Sciences, University of Bristol, Bristol, United Kingdom; 2 Laboratory of Molecular Neuroendocrinology, Department of Biophysics, Paulista School of Medicine, Federal University of São Paulo, São Paulo, Brazil

**Keywords:** Magnocellular neurones, Cell body, Axonal terminal, Proteome, Phosphoproteome, Cytoskeleton, Synapse

## Abstract

The cell bodies of hypothalamic magnocellular neurones are densely packed in the hypothalamic supraoptic nucleus, whereas their axons project to the anatomically discrete posterior pituitary gland. We have taken advantage of this unique anatomical structure to establish proteome and phosphoproteome dynamics in neuronal cell bodies and axonal terminals in response to physiological stimulation. We have found that proteome and phosphoproteome responses to neuronal stimulation are very different between somatic and axonal neuronal compartments, indicating the need of each cell domain to differentially adapt. In particular, changes in the phosphoproteome in the cell body are involved in the reorganization of the cytoskeleton and in axonal terminals the regulation of synaptic and secretory processes. We have identified that prohormone precursors including vasopressin and oxytocin are phosphorylated in axonal terminals and are hyperphosphorylated following stimulation. By multiomic integration of transcriptome and proteomic data, we identify changes to proteins present in afferent inputs to this nucleus.

## Highlights

• Proteomic and phosphoproteomic investigation of distinct neuron compartments.
• Axonal and somatic compartments respond uniquely to physiological stimulation.
• Phosphorylation changes remodel the somatic cytoskeleton and the axonal presynapse.
• Hormone precursors become hyperphosphorylated in response to stimulation.

## Graphical Abstract

**Figure:**
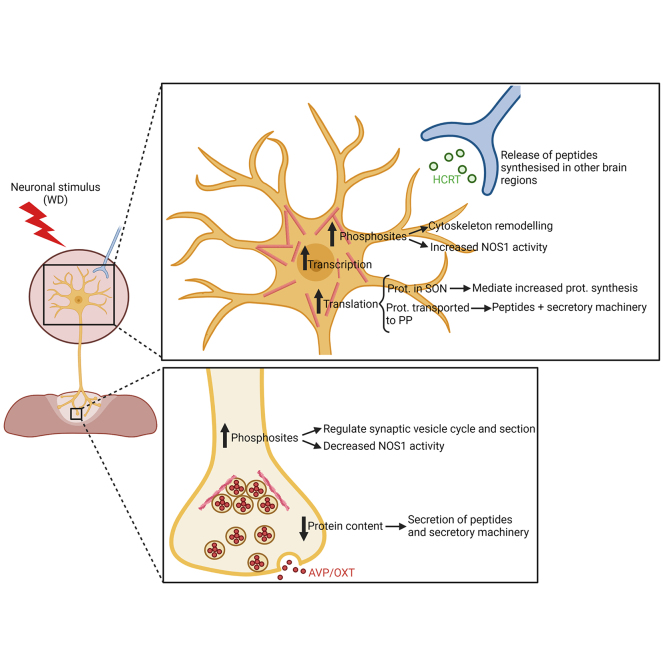

## In Brief

In this work, we have explored the proteomes and phosphoproteomes of somatic neuronal compartments in the supraoptic nucleus of the hypothalamus and their axonal projections in the hypophysis under basal and stimulated conditions. Through comparisons with corresponding transcriptomes of the supraoptic nucleus, we bring a novel perspective to transcriptome, proteome, and phosphoproteome dynamics in this uniquely tractable model neuronal system.

Determining the integrative transcriptome, proteome, and phosphoproteome of a given cell type at any given time is rather challenging, but this is particularly complicated in neurones. It is recognized that neurones have distinct protein populations in their cell bodies and axons (1, 2). Since proteins and their phosphorylation events control cellular function, it is imperative to separately determine the proteomes and phosphoproteomes of cell bodies and axonal terminals to better understand cellular processes in health and disease. The complex projection patterns of neurones make this challenging for most brain nuclei.

Because of its unique anatomical structure, there is one neuronal system that is ideally suited to overcome many of these inherent challenges. The hypothalamo-neurohypophysial system (HNS) is a key neuroendocrine interface that connects the hormonal, neuronal, and vascular systems in all vertebrate species (3, 4). Hormones, such as antidiuretic hormone arginine vasopressin (AVP) and oxytocin (OXT), are made by densely packed populations of magnocellular neurones (MCNs) predominantly located in the hypothalamic supraoptic nucleus (SON) and also the paraventricular nucleus (PVN). In the rat SON, neuronal elements [which comprise 94% of MCNs and only 6% of interneurons (5)] occupy about 74% and glial elements about 8% of the total volume (6). MCNs have one long axon that terminates in the posterior lobe of the pituitary gland (PP) and collaterals that project into other regions of the brain (7). The PP is composed of pituicytes, approximately 30% by volume, and by axonal terminals that comprise 42% of the PP volume with up to 36 million nerve terminals and swellings, based on the estimates of 18,000 MCNs each with one long axon giving rise to an estimated 2000 nerve terminals and swellings (8, 9). Each axon terminal contains ∼260 dense core vesicles (DCVs) packed with peptide hormones, including AVP and OXT, which are destined for release into the blood (9).

When the HNS is osmotically stimulated, such as evoked by water deprivation (WD), there is an increase in hormone release from the PP into the blood as the nerve endings are depolarized (10). AVP MCNs undergo phasic firing that allows action potential broadening and prevents the secretory fatigue caused by prolonged continuous stimulation, optimizing secretion at axon terminals (11). Chronic osmotic stimulation of MCNs results in a striking functional remodeling of the HNS through several activity-dependent changes in the morphology, electrical properties, and biosynthetic and secretory activity of the SON (12, 13, 14), which contribute to the facilitation of hormone synthesis, vesicle transportation, and secretion. While this plasticity has been explored extensively at the transcriptome level (15, 16, 17, 18, 19, 20), dynamic changes in the proteome and phosphoproteome have not thus far been addressed.

In this work, we explore the proteomes and phosphoproteomes of the SON and the neurointermediate lobe (NIL) of the pituitary gland under basal and WD conditions. By integrating transcriptome catalogs, we comprehensively describe dynamic biosynthetic and secretory strategies that fuel the outputs of MCNs.

## Experimental Procedures

### Experimental Design and Statistical Rationale

Six SON and NIL control samples and six SON and NIL WD samples were processed for proteomic and phosphoproteomic analyses as this number of replicates has been proven to provide sufficient statistical power for most proteins with changes >2 fold (21).

### Animals

All experimental procedures involving animals were performed in strict accordance with the provision of the UK Animals (Scientific Procedures) Act (1986). The study was carried out under a Home Office UK licence (PPL PP9294977) and all the protocols were approved by the University of Bristol Animal Welfare and Ethical Review Board. The present study was designed following the ARRIVE (Animal Research: Reporting of *In Vivo* Experiments) guidelines (22).

Twelve male Sprague Dawley rats weighing 250 to 300 g were purchased from Envigo (RRID:RGD_70508) and acclimatized for 10 days. Rats were maintained under a 12:12 light dark cycle (lights on 8.00 AM) at a constant temperature of 21 to 22 °C and a relative humidity of 40%-50% with *ad libitum* access to food and water. Rats were housed in groups of three with environmental enrichment consisting of nesting material, cardboard tube, and a chew block. Animal cages were randomly assigned to control or WD groups. For the WD group, water was removed for 48 h with *ad libitum* access remaining for controls. Forty-eight hours WD has been previously shown to decrease AVP content in the PP by 50% (23).

For proteomic analyses and Western blotting, rats were killed by striking the cranium. The brain was removed from the cranium and placed in an ice-cold brain matrix to separate the forebrain from the hindbrain. The pituitary gland was removed from the base of the cranium and the NIL (containing the PP and the intermediate lobe) dissected from the anterior pituitary. The posterior lobe was not isolated from the intermediate lobe to avoid different levels of intermediate lobe contamination across the posterior lobe samples and to decrease the collection sample time that affects protein phosphorylation (24). Forebrains and NIL were immediately frozen in powdered dry ice and stored at −80 °C. For immunohistochemistry analyses, rats were deeply anesthetized with intraperitoneal administration of sodium pentobarbitone (100 mg/kg,) and transcardially perfused with 0.1 M PBS pH 7.4 followed by 4% (w/v) paraformaldehyde in PBS. The brain and pituitary gland were removed, postfixed in 4% (w/v) paraformaldehyde overnight, and cryoprotected in 30% (w/v) sucrose in PBS prior to freezing the tissues over liquid nitrogen. All sample collections were performed between 9.00 AM and 12.00 PM.

### Protein Extraction for Proteomic and Phosphoproteomic Analyses

SON samples were collected bilaterally using a 1-mm micropunch (Fine Scientific Tools) from 100 μm brain coronal sections in a cryostat as described (25). Proteins from SON and NIL samples were extracted in lysis buffer containing 50 mM Tris–HCl, pH 7.6; 150 mM NaCl; 0.1% (w/v) SDS; 0.5% (w/v) sodium deoxycholate; 1% (v/v) Nonidet P-40; 1 mM EDTA containing the protease inhibitors 1 mM PMSF (Merck, P7626), Pierce Protease Inhibitor Tablets (Thermo Fisher Scientific, A32963) and Pierce Phosphatase Inhibitor Mini Tablets (Thermo Fisher Scientific, A32957) in three sonication cycles of 12 s. Samples were then incubated in ice for 30 min, vortexing every 5 min, and then centrifuged at 10,000 g for 20 min at 4 °C. The supernatant was transferred to a fresh tube and protein concentrations were determined by the Bradford assay.

### TMT Labeling, High pH Reversed-Phase Chromatography, and Phosphopeptide Enrichment

Total proteome and phosphoproteome analysis were performed at the Bristol Proteomics Facility, University of Bristol. Aliquots of 100 μg of each sample were reduced (10 mM TCEP, 55 °C for 1 h), alkylated (18.75 mM iodoacetamide, room temperature (RT) for 30 min), digested with trypsin (2.5 μg trypsin per 100 μg protein; 37 °C, overnight), labeled with Tandem Mass Tag (TMTpro) 16 plex reagents according to the manufacturer’s protocol (Thermo Fisher Scientific, Loughborough, LE11 5RG), and the labeled samples pooled.

For the total proteome analysis, an aliquot of 50 μg of the pooled sample was desalted using a SepPak cartridge according to the manufacturer’s instructions (Waters). Eluate from the SepPak cartridge was evaporated to dryness and resuspended in buffer A (20 mM ammonium hydroxide, pH 10) prior to fractionation by high pH reversed-phase chromatography using an Ultimate 3000 liquid chromatography system (Thermo Fisher Scientific). In brief, the sample was loaded onto an XBridge BEH C18 Column (130 Å, 3.5 μm, 2.1 mm X 150 mm, Waters) in buffer A and peptides eluted with an increasing gradient of buffer B (20 mM ammonium hydroxide in acetonitrile, pH 10) from 0 to 95% (w/v) over 60 min. The resulting fractions (20 in total) were evaporated to dryness and resuspended in 1% (v/v) formic acid prior to analysis by nano-LC MS/MS using an Orbitrap Fusion Lumos mass spectrometer (Thermo Fisher Scientific).

For the phosphoproteome analysis, the remainder of the TMT-labeled pooled sample was desalted using a SepPak cartridge (Waters). The eluate from the SepPak cartridge was evaporated to dryness and subjected to TiO2-based phosphopeptide enrichment according to the manufacturer’s instructions (Pierce). The flow-through and washes from the TiO2-based enrichment were then subjected to FeNTA-based phosphopeptide enrichment according to the manufacturer’s instructions (Pierce). The phospho-enriched samples were again evaporated to dryness and then resuspended in 1% formic acid prior to analysis by nano-LC MSMS using an Orbitrap Fusion Lumos mass spectrometer (Thermo Fisher Scientific).

### Nano-LC Mass Spectrometry

High pH RP fractions (Total proteome analysis) or the phospho-enriched fractions (phosphoproteome analysis) were further fractionated using an Ultimate 3000 nano-LC system in line with an Orbitrap Fusion Lumos mass spectrometer (Thermo Fisher Scientific). In brief, peptides in 1% (v/v) formic acid were injected onto an Acclaim PepMap C18 nano-trap column (Thermo Fisher Scientific). After washing with 0.5% (v/v) acetonitrile, 0.1% (v/v) formic acid peptides were resolved on a 250 mm × 75 μm Acclaim PepMap C18 reverse phase analytical column (Thermo Fisher Scientific) over a 150 min organic gradient, using seven gradient segments (1–6% solvent B over 1 min, 6–15% B over 58 min, 15–32% B over 58 min, 32–40% B over 5 min, 40–90% B over 1 min, held at 90% B for 6 min, and then reduced to 1% B over 1 min) with a flow rate of 300 nl min^−1^. Solvent A was 0.1% (v/v) formic acid and solvent B was aqueous 80% (v/v) acetonitrile in 0.1% (v/v) formic acid. Peptides were ionized by nano-electrospray ionization at 2.0 kV using a stainless-steel emitter with an internal diameter of 30 μm (Thermo Fisher Scientific) and a capillary temperature of 300 °C.

All spectra were acquired using an Orbitrap Fusion Lumos mass spectrometer controlled by Xcalibur 3.0 software (https://www.thermofisher.com/order/catalog/product/es/en/OPTON-30965) (Thermo Fisher Scientific) and operated in data-dependent acquisition mode using an SPS-MS3 workflow. FTMS1 spectra were collected at a resolution of 120,000, with an automatic gain control (AGC) target of 200,000 and a max injection time of 50 ms. Precursors were filtered with an intensity threshold of 5000, according to charge state (to include charge states 2–7) and with monoisotopic peak determination set to peptide. Previously interrogated precursors were excluded using a dynamic window (60s ± 10 ppm). The MS2 precursors were isolated with a quadrupole isolation window of 0.7 m/z. ITMS2 spectra were collected with an AGC target of 10,000, max injection time of 70 ms, and collision-induced dissociation energy of 35%.

For FTMS3 analysis, the Orbitrap was operated at 50,000 resolution with an AGC target of 50,000 and a max injection time of 105 ms. Precursors were fragmented by high energy collision dissociation at a normalized collision energy of 60% to ensure maximal TMT reporter ion yield. Synchronous Precursor Selection was enabled to include up to 10 MS2 fragment ions in the FTMS3 scan.

### Data Processing

The raw data files were processed and quantified using Proteome Discoverer software v2.4 (https://www.thermofisher.com/es/es/home/industrial/mass-spectrometry/liquid-chromatography-mass-spectrometry-lc-ms/lc-ms-software/multi-omics-data-analysis/proteome-discoverer-software.html) (Thermo Fisher Scientific) and searched against the UniProt Rat database (downloaded July 2021: 35,859 entries) using the SEQUEST HT algorithm. Peptide precursor mass tolerance was set at 10 ppm, and MS/MS tolerance was set at 0.6 Da. Search criteria included oxidation of methionine (+15.995 Da), acetylation of the protein N-terminus (+42.011 Da), and Methionine loss plus acetylation of the protein N-terminus (−89.03 Da) as variable modifications and carbamidomethylation of cysteine (+57.0214) and the addition of the TMTpro mass tag (+304.207) to peptide N-termini and lysine as fixed modifications. For the phosphoproteome analysis, phosphorylation of serine, threonine, and tyrosine (+79.966) was also included as a variable modification. Searches were performed with full tryptic digestion and a maximum of two missed cleavages were allowed. The reverse database search option was enabled, and all data (at the peptide and protein level) was filtered to satisfy false discovery rate of 5%.

### Protein Abundance Processing

Protein groupings were determined by PD2.4, where proteins within each group with equal quantification and identification metrics were termed candidate masters by the software. The master protein selection was improved with an in-house script, which retrieved annotation information for all candidate master proteins and selected master proteins for each group based upon annotation quality. This enabled us to infer biological trends more effectively in the dataset without any loss in the quality of identification or quantification. The MS data were searched against the Rat Uniprot database retrieved July 2021 and updated with additional annotation information added on 2022-02-08. The protein abundances were normalized within each sample to total peptide amount, then Log2 transformed to bring them closer to a normal distribution.

### Phosphopeptide Abundance Processing

The phosphorylation status of identified peptide spectral matches was determined by PD2.4 and the site of phosphorylation predicted by PD2.4 using the PhosphoRS module. Phosphorylation sites predicted by PhosphoRS with greater than 70% confidence were taken as the likely phosphorylation site, and peptide spectral matches with identical sequences and predicted phosphorylation sites were grouped to provide improved quantitation and confidence.

We performed two separate analyses: one to compare the total protein levels across the samples and a second in which we included a phosphopeptide enrichment step to allow us to compare phosphopeptide levels across the same samples. As such, the normalization factor for the total protein dataset was applied to the phospho dataset as it provided a more useful normalization metric than total phosphopeptide abundance, which is more likely to vary based upon the biological conditions of interest. Where possible, phosphopeptides were matched to corresponding proteins in the total protein dataset and the Log2 Normalized Protein abundance was subtracted from the Log2 Normalized Phosphopeptide abundance to adjust the phosphopeptide abundance for any changes in the total protein level. Statistical significance was then determined using Welch’s t-tests between the conditions of interest. Since it has been discussed that the use of multiple testing–corrected false discovery rate maybe too blunt and restrictive for proteomic analysis (26), especially when analyzing such a heterogeneous and complex tissue as the brain (27, 28, 29, 30, 31, 32), we considered uncorrected *p* < 0.05 as differentially produced proteins and phosphosites in all our SON and NIL analysis. All data have been deposited at the ProteomeXchange Consortium *via* the PRIDE partner repository (33) with the dataset identifier PXD040870.

### Data Analysis

Principal component analysis (PCAs) were calculated using the FactoMineR package and then plotted using the ggplot2 package. Principal components 1 and 2 were plotted against each other to give an indication of the main sources of variance, and principal components 3 and 4 were plotted to infer any further trends.

In volcano plots, for each comparison, the -log10 *p*-value of each protein was plotted against the log2 fold change (Log2FC) using GraphPad Prism 8.4.3. Proteins where *p* < 0.05 are highlighted in blue and red.

Due to the lack of published data describing the single-cell transcriptome of the rat SON MCNs, we used data from mouse PVN single-cell RNA-seq (34) to gain insight into proteomic and phosphoproteomic changes of MCN compartments during WD. Seurat 4.1.3 (35) was used to reanalyze the data available at the Gene Expression Omnibus under accession code GSE148568 and then plotted using the Seurat and ggplot2 package. By using the genes *Avp/Oxt* and *Caprin2* as markers (36), we have identified and integrated 366 MCNs from four clusters to generate a single list of genes (21,917) expressed in this neuronal population (supplemental Fig. S1, *A*–*D*). By applying a cut-off of normalized counts >0.5, we obtained a list of the top 10.6% expressed genes in the MCNs (2218). This list was used to infer the proteomic and phosphoproteomic changes in these neurons. In the SON, the genes encoding 1694 proteins were among the 10% most enriched genes in MCNs, 132 and 76 of which changed their total content and phosphorylation state, respectively, in response to WD. In the NIL, the genes encoding 1721 proteins were among the 10% most enriched genes in MCNs, 209 and 217 of which changed their total content and phosphorylation state, respectively, in response to WD.

Gene Ontology (GO) gene set enrichment analysis was performed using the gProfiler2 package (37) in R (R core team, 2021, version 4.0.3, https://www.R-project.org/) using a significance threshold of <0.05 Padj for enriched terms. A background list of the 10% top genes expressed in MCNs was used with exception of the SON phosphoproteome that did not retrieve any results, so all rat annotated genes were used as background. Databases searched included GO:Cellular Component (GO:CC), GO:Molecular Function (GO:MF), and GO:Biological Process (GO:BP) and Kyoto Encyclopedia of Genes and Genomes (KEGG). Ontologies for the synapse were performed using SynGO (38).

The phosphorylation state change (ΔPs) value calculation was adapted from (39). Briefly, the ΔPs values for protein isoforms encoded by the same gene were determined by the sum of Log2FC of all phosphorylated peptides with statistically significant changes (*p* < 0.05) in both SON and NIL when comparing the WD and control groups in the phosphoproteome data. We next calculated the average SD of adjusted log2 normalized abundance from all identified phosphopeptides in the SON (SD = 0.17) and NIL (SD = 0.2). We applied a cut-off at ±0.34 for the SON and ±0.40 for the NIL (>2 SD) to represent the cumulative protein phosphorylation, determining the hyperphosphorylated and hypophosphorylated phosphoproteins on those tissues in response to WD.

We classified changes in the proteome and ΔPs as endogenous peptides, enzymes, G protein-coupled receptors, catalytic receptors, channels, transporters, transcription factors, and other pharmacological targets using the functional classification of the International Union of Basic and Clinical Pharmacology (40) in association with a manually curated list of validated human transcription factors (41). Only the proteins with an existing entry in these databases were cataloged according to this classification. The same approach was used to characterize the newly discovered peptides without or very low mRNA expressed in the SON.

Mapping of phosphosites and changes in phosphorylation to the protein sequence was done by using PhosphoSitePlus (https://www.phosphosite.org).

The relationship between basal transcriptomes and proteomes was examined by comparing the proteome output obtained in the present study in basal conditions with a complete rat transcriptome combining all the genes detected in previous SON transcriptomic analysis in Sprague Dawley (42) and Wistar Han (20) rats in basal conditions. We found that 14.846 genes are commonly expressed in both strains, while only 356 and 545 genes are uniquely expressed in the SON of Wistar Han and Sprague Dawley rats (supplemental Fig. S2). A single list of genes with normalized counts >10 expressed in the SON of Wistar Han and Sprague Dawley rats (15.747 total genes) was used to determine the non-SON–synthesized peptide inputs into both SON and NIL.

### Immunofluorescence

Coronal sections of the forebrain containing the hypothalamus and NILs were cut on a cryostat at 40 μm and kept in PBS at 4 °C. To prevent nonspecific protein binding, sections were blocked in PBS containing 0.3% (v/v) Triton X-100, 4% (w/v) bovine serum albumin (BSA), and 5% (v/v) donkey serum at RT for 1 h. Following this, the NIL sections were incubated overnight at 4 °C with the primary antibodies against NOS1 (1:200, Santa Cruz biotechnology, sc-5302, RRID:AB_626757), phospho-nNOS (Ser852) (1:100, Thermo Fisher Scientific, PA5-38305, RRID:AB_2554906), phospho-Synapsin I (Ser62, Ser67) (1:200, Millipore, AB9848, RRID:AB_673006), phospho-Synapsin 2 (Ser425) (1:200, Thermo Fisher Scientific, PA5-64855, RRID:AB_2663626), and Synapsin (1:200, Cell Signaling Technology, 2312S, RRID:AB_2200102) in PBS containing 0.3% (v/v) Triton X-100, 4% (w/v) BSA, and 1% (v/v) donkey serum. Tissue sections containing the SON were incubated with antibodies against NOS1 (1:200, Santa Cruz biotechnology, sc-5302, RRID:AB_626757), Orexin A/Hypocretin-1 (1:1000, R and D Systems, MAB763, RRID:AB_2117627) Phospho-JUND (Ser255) (1:100, Thermo Fisher Scientific, PA5-104821, RRID:AB_2816294), Phospho-nNOS (Ser1417) (1:500, Thermo Fisher Scientific, PA1-032, RRID:AB_32502), Phospho-Stathmin 1 (Ser24) (1:200, MyBioSource, MBS9600965, RRID: AB_2910204), Phospho-S6 Ribosomal Protein (Ser240/244) (1:500, Cell Signaling Technology, 5364S, RRID:AB_10694233), Stathmin 1 (1:500, GeneTex, GTX104707, RRID:AB_1241361), and S6 Ribosomal Protein (1:100, Cell Signaling Technology 2317S, RRID:AB_2238583) in PBS containing 0.3% (v/v) Triton X-100, 4% (w/v) BSA, and 1% (v/v) donkey serum. All antibodies were incubated at 4 °C overnight, with the exemption of the antibodies against Orexin A/Hypocretin-1 and Phospho-Stathmin 1 (Ser24) that were incubated for 48 h. Following incubation with the primary antibodies, sections were washed in PBS and incubated with the secondary antibodies donkey anti-rabbit Alexa Fluor Plus 488 (Thermo Fisher Scientific, A32790, RRID:AB_2762833) and donkey anti-mouse Alexa Fluor 594 (Molecular Probes, A-21203, RRID:AB_141633) at a 1:500 dilution in PBS containing 0.1% (v/v) Triton, 4% (w/v) BSA, and 1% (v/v) donkey serum at RT for 1 h. Then, the sections were washed in PBS, incubated with 4′,6-diamidino-2-phenylindole dihydrochloride (D1306; Molecular Probes) in PBS, and mounted with ProLong Gold Antifade Mountant (Thermo Fisher Scientific, P36930).

Images were acquired using a Leica SP5-II AOBS confocal laser scanning microscope attached to a Leica DMI 6000 inverted epifluorescence microscope with a 20× and a 63× PL APO CS lens. Raw image files were processed to generate composite images using the open access image analysis software, Fiji (https://fiji.sc/).

### Western Blot

Protein extraction from the NIL was performed as described (43). Protein samples were prepared to 1 × Laemmli buffer solution (2% w/v SDS, 10% v/v glycerol, 5% v/v 2-mercaptoethanol, 0.002% w/v bromophenol blue, and 0.125 M Tris–HCl, pH 6.8). Samples were heated at 95 °C in a hot block for 5 min. For semiquantitative analysis of protein levels, 20 μg/lane of total protein (determined in duplicate by Bio-Rad Protein Assay with BSA as standards) was loaded for control and WD samples. Proteins were fractionated on 8% (w/v) SDS polyacrylamide gels and transferred to Immobilon-P PVDF Membrane (Merck, IPVH00010). Membranes were incubated in 5% (w/v) BSA in Tris-buffered saline (150 mM NaCl; 20 mM Tris–HCl, pH 7.6) with 0.1% (v/v) Tween 20 (TBS-T) for 1 h followed by incubation with the primary antibodies NOS1 (1:200, Santa Cruz biotechnology, sc-5302, RRID:AB_626757), phospho-nNOS (Ser852) (1:1000, Invitrogen, PA5-38305, RRID:AB_2554906), phospho-Synapsin I (Ser62,Ser67) (1:1000, Merk, AB9848, RRID:AB_673006), phospho-Synapsin 2 (Ser425) (1:5000, Invitrogen, PA5-64855, RRID:AB_2663626), Synapsin (1:1000, Cell Signaling Technology, 2312S, RRID:AB_2200102), and Tubulin (1:5000, Covance, MMS-489P, RRID:AB_10096105) overnight at 4 °C. Following three washes in TBS-T, the membranes were incubated with the appropriate secondary antibody conjugated with horseradish peroxidase for 1 h. After three washes in TBS-T, the signal was detected using chemiluminescence SuperSignal West Dura Extended Duration Substrate reagent (Thermo Fisher Scientific, 34075). Immunoblots were stripped in Restore Western Blot Stripping Buffer (Thermo Fisher Scientific, 21059) and reprobed to assess the multiple proteins in the same blot.

### Statistical Analysis

Statistical analyses were performed with GraphPad 8.4.3 Software (https://www.graphpad.com/updates/prism-843-release-notes). For Western blot signal quantification, assessment of the normality of data was performed by Shapiro–Wilk test. Means between two groups were compared using independent-sample unpaired Student's t tests where data are expressed as box and whisker plots. For the P-SYN1 and P-SYN quantifications, one sample was excluded from the analysis due to the absence of total SYN signal (supplemental Fig. S3), otherwise all samples were included in the analysis. Spearman correlation analysis was also performed in GraphPad Prism Prism 8.4.3. In all cases, *p* < 0.05 was considered statistically significant.

## Results

### Quantitative Proteome and Phosphoproteome of the Rat SON

**Fig. 1:**
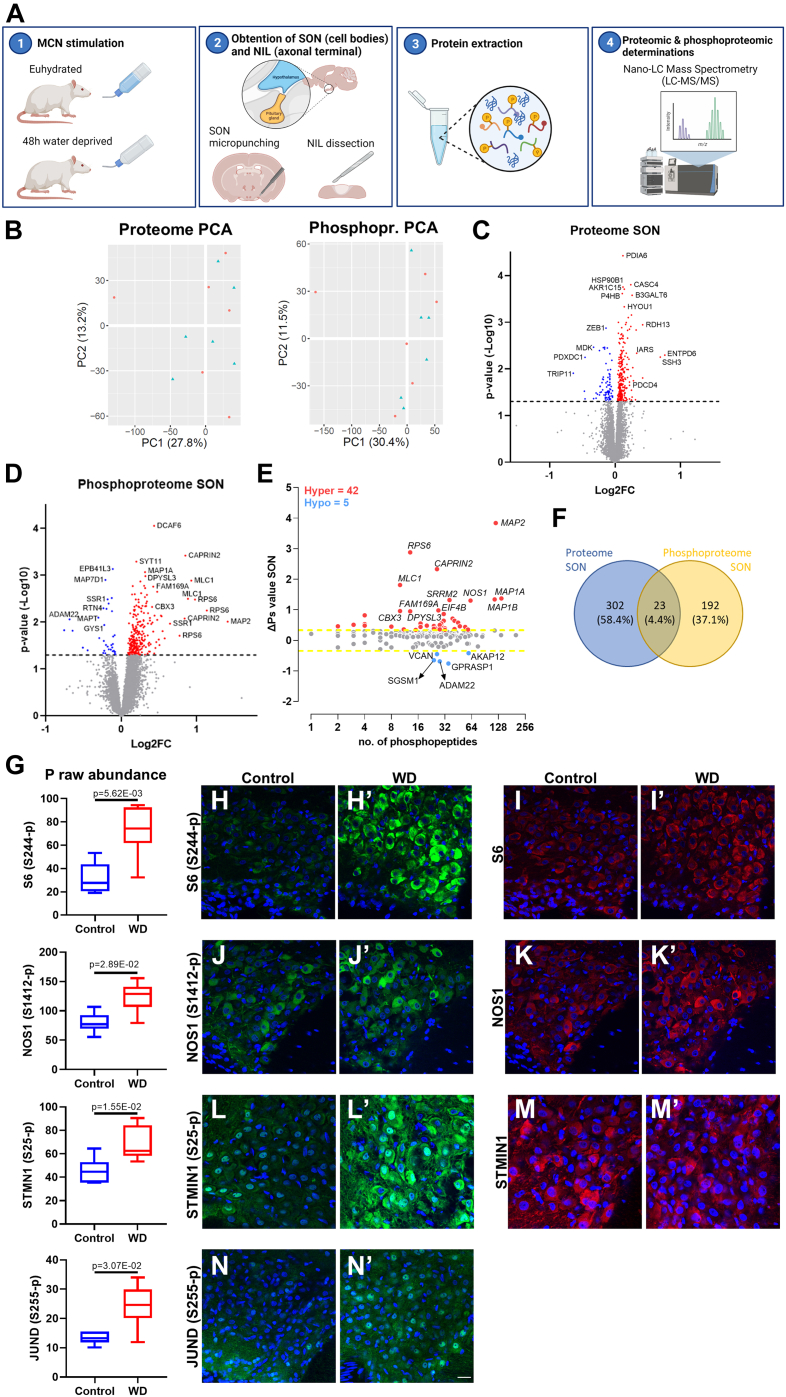
**Quantitative proteome and phosphoproteome of the rat supraoptic nucleus.***A*, graphical representation of the experimental approach (1). Twelve adult Sprague Dawley rats were divided into two groups: six control with constant access to water and six subjected to a 48-h water deprivation protocol (WD) to activate magnocellular neurones (MCNs) (2). The supraoptic nucleus (SON, mainly containing MCN cell bodies and dendrites) was punched from the hypothalamus, and the neurointermediate lobe (NIL, mainly containing axonal terminals from the posterior pituitary as well as the intermediate lobe) was dissected from the anterior pituitary (3). Proteins from SON and NIL were extracted and processed for Nano-LC Mass Spectrometry (LC-MS/MS) (4). Proteomics and phosphoproteomic determinations were performed by LC-MS/MS. Generated using BioRender (https://biorender.com/). *B*, principal component analysis (PCA) of the SON proteome and phosphoproteome in control (*blue*, n = 6) and WD rats (*red*; n = 6). *C*, volcano plot of WD *versus* control SON proteome showing 247 upregulated (*p*-value <0.05, *red*) and 78 downregulated (*p*-value <0.05, *blue*) proteins. *D*, volcano plot of WD *versus* control SON phosphoproteome showing 252 hyperphosphorylation (*p*-value <0.05, *red*) and 36 hypophosphorylation (*p*-value <0.05, *blue*) events. *E*, global overall phosphorylation state change (ΔPs) analysis of phosphoproteins between control and WD rats in the SON. Numbers of hyperphosphorylated (Hyper) and hypophosphorylated (Hypo) peptides are shown. *Dotted lines*, ΔPs = ±0.34. *F*, Venn diagram showing 23 proteins in common with changes at the proteome and phosphoproteome level in response to WD. *G*, phospho raw abundance for S244-p S6, S1412-p NOS1, S25-p STMN1, and S255-p JUND in the SON according to LC-MS/MS between control (n = 6) and WD (n = 6) rats. *H*, immunohistochemistry against S244-p S6 in the SON of control and (H′) WD rats. *I*, immunohistochemistry against S6 in the SON of control and (I′) WD rats. *J*, immunohistochemistry against S1412-p NOS1 in the SON of control and (J′) WD rats. *K*, immunohistochemistry against NOS1 in the SON of control and (K′) WD rats. *L*, immunohistochemistry against S25-p STMN1 in the SON of control and (L′) WD rats. *M*, immunohistochemistry against STMN1 in the SON of control and (M′) WD rats. *N*, immunohistochemistry against S255-p JUND in the SON of control and (N′) WD rats. Images are representative of n = 4. Scale bar represents 25 μm. LC-MS/MS, Nano-LC mass spectrometry.

To stimulate MCNs, animals were WD for 48 h. Stimulated animals were compared to euhydrated controls. The SON (containing MCN cell bodies and dendrites) was punched from the hypothalamus, and the NIL (containing axonal terminals in the PP and the intermediate lobe) was dissected from the anterior lobe of the pituitary. Proteins were extracted and processed for Nano-LC Mass Spectrometry (LC-MS/MS, Fig. 1*A*). A catalog of proteins detected in the SON and NIL and differentially produced proteins and phosphosites between control and WD rats is presented in the supporting information (supplemental Tables S1 and S2).

Proteome analysis of the SON identified 7529 proteins (supplemental Table S1). PCA of proteome and phosphoproteome data did not reveal a separation pattern between control and WD groups (Fig. 1*B*). Of the 7529 proteins detected in the SON, the data indicated 325 differentially abundant proteins in WD SON, with 247 being increased and 78 decreased in content (Fig. 1*C*). We asked about the phosphorylation status of identified proteins. We found 9900 phosphorylated sites, 288 of which were differentially phosphorylated in response to WD (*p*-value <0.05). Two hundred fifteen proteins underwent changes in phosphorylation in response to WD (*p*-value <0.05), which included 252 hyperphosphorylation and 36 hypophosphorylation events (Fig. 1*D*). We also globally quantified changes in phosphorylation in order to obtain single values for changes in the overall phosphorylation status of individual proteins. This analysis measured the overall ΔPs of each protein as described by Wang *et al*. (2018). In the SON, the ΔPs showed that 42 proteins were significantly hyperphosphorylated (ΔPs >0.34), while five were hypophosphorylated (ΔPs < −0.34) (Fig. 1*E*). The top three ΔPs hyperphosphorylated proteins were MAP2, S6, and Cytoplasmic activation/proliferation-associated protein 2 (CAPRIN2), and the hypophosphorylated proteins were G protein coupled receptor–associated sorting protein 1 (GPRASP1), Disintegrin and metalloproteinase domain-containing protein 22 (ADAM22), and Glycogen synthase (GYS1). Only 23 proteins that changed in overall content also had phosphorylation modifications in response to WD (Fig. 1*F*).

The phosphoproteome data was validated using commercially available phospho-antibodies against significantly altered phosphosites in the control and WD SON. We investigated phosphosites for 40S ribosomal protein S6 (S6; S244-p) that showed a Log2FC of 1.13 (*p*-value = 5.62E-03), nitric oxide synthase (NOS1, S1412-p) with a Log2FC of 0.36 (*p*-value = 2.89E-02), STATHMIN 1 (STMN1, S25-p) with a Log2FC of 0.55 (*p*-value = 1.55E-02), and JUND (S255-p) with a Log2FC of 0.51 (*p*-value = 3.07E-02) (Fig. 1*G*). In addition, we immunolabeled S6, NOS1, and STMN1 proteins. Immunohistochemical studies supported increased phosphorylation of S6 S244-p in MCNs (Fig. 1*H*, H’) with no change to S6 protein content (Fig. 1*I*, I’). The phosphorylation of the S1412-p residue of NOS1 (Fig. 1*J*, J’) appeared to increase in a population of MCNs that produce NOS1 (Fig. 1*K*, K’). Immunostaining against S25-p STMN1 detected the presence of this phospho residue in the SON and revealed increased staining in MCNs in response to WD (Fig. 1*L*, L’). No differences were observed in immunostaining of the STMN1 protein (Fig. 1*M*, M’). Immunostaining of S255-p JUND revealed the presence of this phospho residue in the SON and confirmed an increase in phosphorylation in MCNs in WD (Fig. 1*N*, N’). We have previously reported increased JUND protein content by Western blot and immunohistochemistry in the WD SON (44). All these findings agreed with the LC-MS/MS output.

### Quantitative Proteome and Phosphoproteome of the Rat NIL

**Fig. 2:**
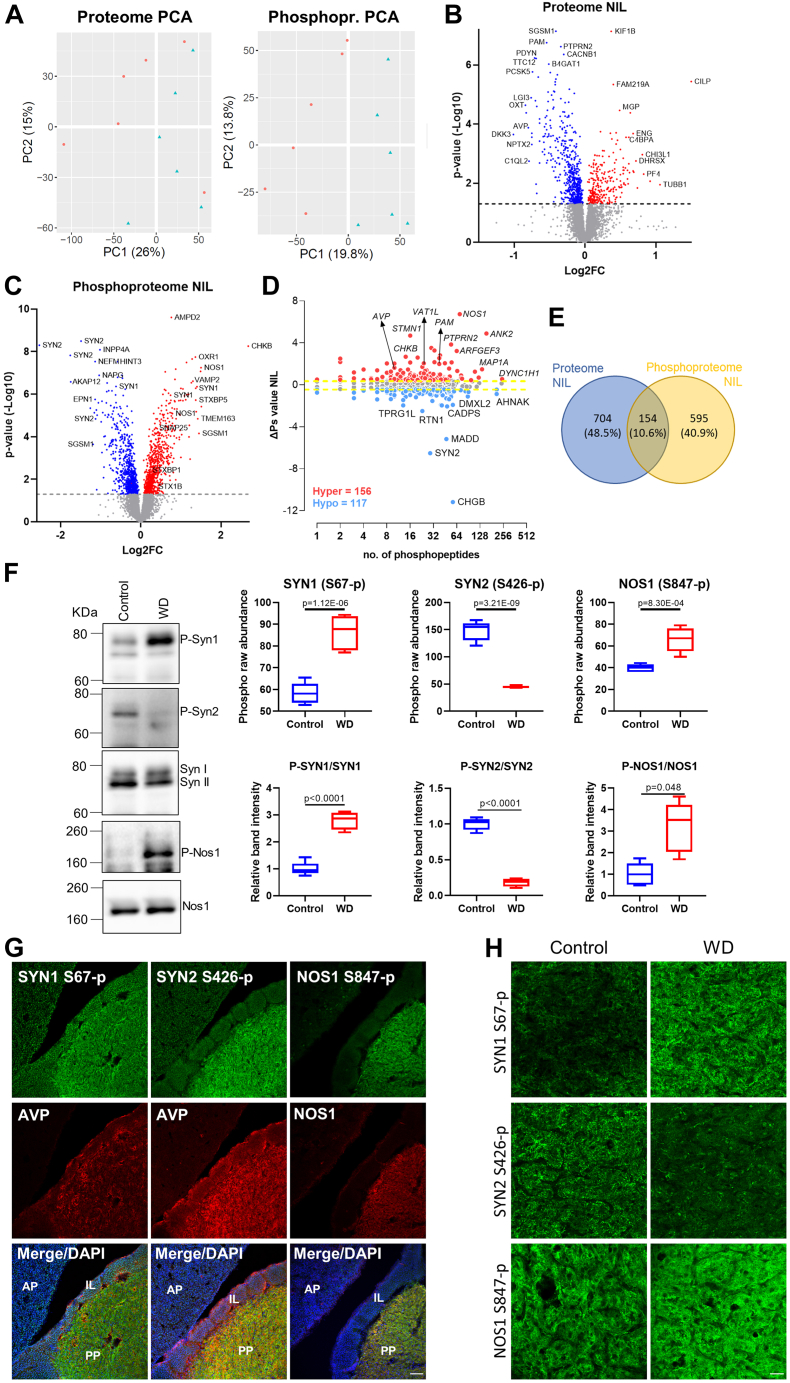
**Quantitative proteome and phosphoproteome of the rat neurointermediate lobe.***A*, principal component analysis (PCA) of the NIL proteome and phosphoproteome in control (*blue*, n = 6) and water-deprived (WD) rats (*red*; n = 6). *B*, volcano plot of WD *versus* control NIL proteome showing 276 upregulated (*p*-value <0.05, *red*) and 573 downregulated (*p*-value <0.05, *blue*) proteins. *C*, volcano plot of WD *versus* control NIL phosphoproteome showing 746 hyperphosphorylation (*p*-value <0.05, *red*) and 755 hypophosphorylation (*p*-value <0.05, *blue*) events. *D*, global ΔPs analysis of phosphoproteins between control and WD rats in the NIL. Numbers of hyperphosphorylated (Hyper) and hypophosphorylated (Hypo) peptides are shown. *Dotted lines*, ΔPs = ±0.4. *E*, Venn diagram showing 151 proteins in common with changes at the proteome and phosphoproteome level in response to WD. *F*, phospho raw abundance for S67-p SYN1, S426-p SYN2, and S426-p SYN2 in the NIL according to LC-MS/MS between control (n = 6) and WD (n = 6) rats. Western blotting analysis of S67-p SYN1 (normalized against SYN1), S426-p SYN2 (normalized against SYN2), and S847-p NOS1 (normalized against NOS1) in control (n = 5) and WD NILs (n = 5 for S847-p NOS1 and n = 4 for S67-p SYN1 and S426-p SYN2). *G*, immunohistochemistry against S67-p SYN1 and arginine vasopressin (AVP), S426-p SYN2 and AVP, and S847-p NOS1 and NOS1 in the pituitary gland of control rats showing the anterior pituitary (AP), intermediate lobe (IL), and posterior pituitary (PP). Images are representative of n = 4. Scale bar represents 75 μm. *H*, immunohistochemistry against S67-p SYN1, S426-p SYN2, and S847-p NOS1 in the PP of control and WD rats. Images are representative of n = 4. Scale bar represents 25 μm. ΔPs, phosphorylation state change; LC-MS/MS, Nano-LC mass spectrometry; NIL, neurointermediate lobe.

Proteome analysis identified 8174 proteins in the NIL (supplemental Table S2). PCA using all proteins revealed distinct separation between the total proteomes of control and WD samples with PC1 accounting for 26% of total variance between samples, which was attributable to WD (Fig. 2*A*). PCA also displayed a distinctive separation between the phosphoproteomes of control and WD samples with PC1 explaining 19.8% of the total variance between samples that was attributable to the experimental condition (Fig. 2*A*). Out of 8174 total proteins, 858 changed their protein content as a consequence of WD (*p*-value <0.05) with 577 proteins decreasing their total content and 281 increasing their total protein content (Fig. 2*B*). We again asked about the phosphorylation status of identified proteins. We found 9130 phosphorylation sites, 1501 of which were differentially phosphorylated in response to WD (*p*-value <0.05). Seven hundred forty-nine proteins underwent changes in phosphorylation (*p*-value <0.05), which included 746 hyperphosphorylation and 755 hypophosphorylation events (Fig. 2*C*). ΔPs analysis revealed 156 significant hyperphosphorylated (ΔPs >0.4) proteins and 117 hypophosphorylated proteins (ΔPs <0.4) (Fig. 2*D*). The top three ΔPs hyperphosphorylated proteins were NOS1, STMN1, and ANK2, while the hypophosphorylated ones included Secretogranin-1 (CHGB), SYN2, and MAP kinase-activating death domain protein (MADD). A total of 151 proteins with alterations to their overall content also underwent changes in phosphorylation in WD (Fig. 2*E*).

NIL phosphoproteome data was validated by Western blot and immunohistochemistry. We used commercially available phospho-antibodies against the phosphosites of Synapsin 1 (SYN1, S67-p) that showed a Log2FC of 0.74 (*p*-value = 1.12E-06), Synapsin 2 (SYN2, S426-p) with a Log2FC of −1.49 (*p*-value = 3.21E-09), and NOS1 (S847-p) with a Log2FC of 0.67 (*p*-value = 8.30E-04) (Fig. 2*F*), as well as antibodies against SYN and NOS1 proteins. Immunoblotting confirmed an increase in SYN1 S67-p, decrease in SYN2 S426-p, and increased NOS1 S847-p following WD (Figs. 2*F* and S3). Immunostaining showed the location of these phosphosites predominantly in the PP which was marked with AVP (Fig. 2*G*). Higher magnification images of the PP in WD confirmed an increase in SYN1 S67-p, a decrease in SYN2 S426-p, and an increase in NOS1 S847-p (Fig. 2*H*). Overall, the pattern of phosphoregulation observed in the SON and NIL agreed with the LC-MS/MS output.

### Pathway Analyses and Functional Classification of the Proteomes and Phosphoproteomes of SON and NIL

**Fig. 3:**
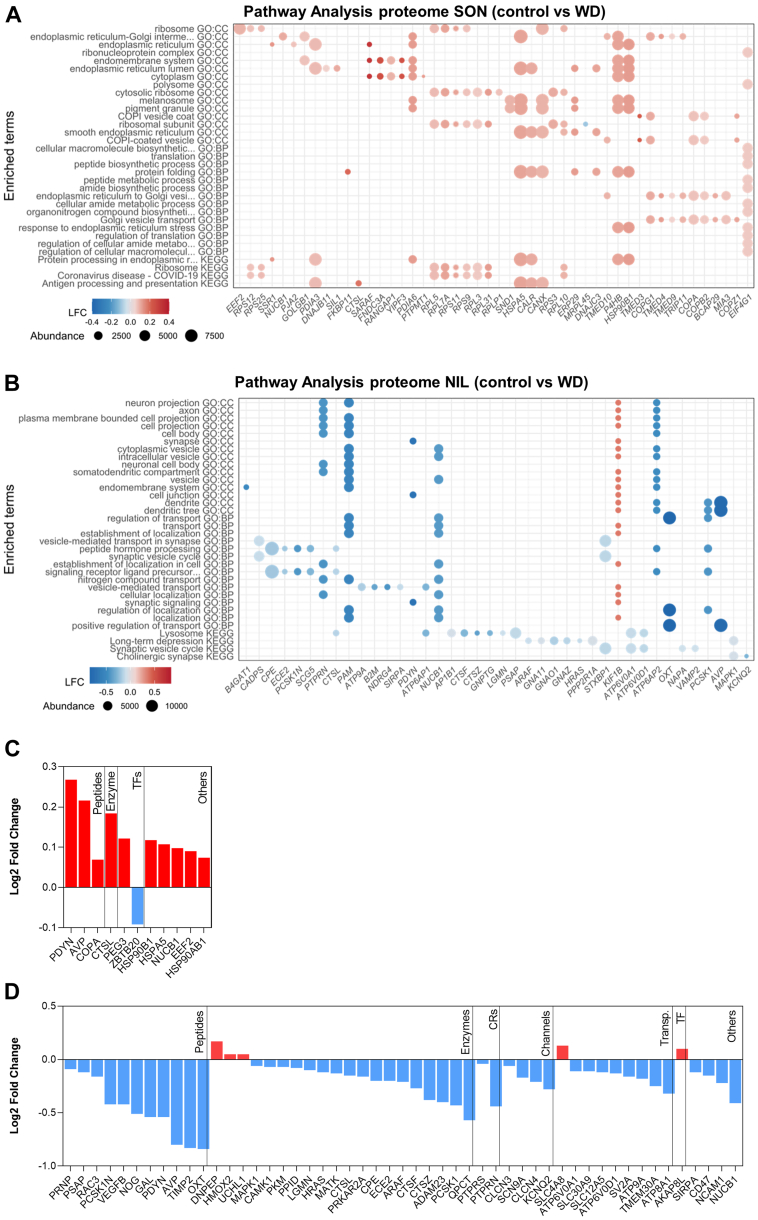
**Pathway analyses and functional classification of the proteomes of supraoptic nucleus and neurointermediate lobe.***A*, pathway analysis of changes in the SON proteome as a result of water deprivation (WD) using GO and KEGG databases. Dot plot of up to 15 enriched terms retrieved for each category ranked according to P_Adj_ value from top to bottom in increasing order. The top 10 most significant associated differentially expressed proteins of each overrepresented category are shown as dots colored based on Log2FC and sized according to total normalized protein expression following WD. *B*, pathway analysis of changes in the NIL proteome as a result of WD using GO and KEGG databases. Dot plot of up to 15 enriched terms retrieved for each category ranked according to P_Adj_ value from top to bottom in increasing order. The top 10 most significant associated differentially expressed proteins of each overrepresented category are shown as dots colored based on Log2FC and sized according to total normalized protein expression following WD. *C*, proteome Log2FC changes in the rat SON as a consequence of WD categorized according to their pharmacological classification or their function as a transcription factor. *D*, proteome Log2FC changes in the rat NIL as a consequence of WD categorized according to their pharmacological classification or their function as a transcription factor. GO, gene ontology; KEGG, Kyoto Encyclopedia of Genes and Genomes; NIL, neurointermediate lobe; Log2FC, Log2 fold change; SON, supraoptic nucleus.

To gain functional insights into the responses of the MCN compartments localized in the SON and NIL to WD, we performed pathway analysis of the differential produced proteins between control and WD samples by interrogating GO and KEGG databases (supplemental Fig. S4). To be more informative of protein changes occurring specifically within the MCN cell population in WD, we selected MCN-enriched proteins for analysis based on the top 10% of abundant protein-encoding genes expressed in MCNs. Briefly, single-cell data from mouse PVN [59] was reanalyzed to identify MCNs, in order to obtain the top 10% genes expressed in MCNs (supplemental Fig. S1). We show enriched terms (restricted to up to 15 terms) retrieved for each category ranked according to P_Adj_ value (Fig. 3). In addition, we plotted the 10 topmost significant associated differentially produced proteins colored based on Log2FC and sized according to total normalized protein following WD. In the SON, the terms retrieved for the GO:CC, GO:MF, and GO:BP categories highlighted terms associated with translation and protein synthesis (Fig. 3*A* and supplemental Table S3). In addition, overrepresentation analysis by KEGG identified “Protein processing in endoplasmic reticulum” as an enriched pathway (Fig. 3*A* and supplemental Table S3). Strikingly, most of the top regulated proteins increased their content in response to WD. Amongst the most abundant proteins following WD were a series of chaperones known to regulate protein folding and stress in the endoplasmic reticulum including the heat shock protein family A (Hsp70) Member 5 (HSPA5), also known as endoplasmic reticulum chaperone BiP, protein disulfide-isomerase (P4HB), and calreticulin (CALR) (45).

In the NIL, terms highlighted in the GO:CC category included “synapse” and “vesicle”, among others (Fig. 3*B* and supplemental Table S3). The GO:BP included several terms related to synaptic signaling, vesicle-regulated transport, and secretion (Fig. 3*B* and supplemental Table S3). KEGG analysis identified the enriched pathways “Synaptic vesicle cycle” and “Cholinergic synapse” among others (Fig. 3*B* and supplemental Table S3). No terms were retrieved for the GO:MF category. Some of the proteins with biggest Log2FC included the peptides AVP and OXT and the enzyme peptidylglycine alpha-amidating monooxygenase (PAM) that mediates C-amidation of endogenous peptides including AVP and OXT (46). Other proteins with large Log2FC were those involved in synaptic vesicle release peptides such as calcium-dependent secretion activator 1 (CADPS) and syntaxin-binding protein 1 (STXBP1) (47). Altogether, these data suggests that changes in the proteome in response to WD in the SON are associated with protein synthesis, while in the NIL, they are related to synaptic signaling and transport.

To further investigate differentially produced proteins in separate compartments of the HNS, we mined the IUPHAR (40) and a transcription factor database (41) to report physiological and pharmacological classifications or identity as transcription factors. In addition to classifying all the proteins that changed their content in response to WD (supplemental Fig. S5), we focused on the classification of those proteins that, as well as changing their content as a result of WD, the genes encoding these proteins are among the 10% most abundant genes in MCN. In the SON, we identified the peptides AVP and proenkephalin-B (PDYN), the enzyme cathepsin L1 (CTSL), and a series of heat shock proteins including heat shock protein 90 beta family member 1 (HSP90B1), heat shock protein family A (Hsp70) member 5 (HSPA5), and heat shock protein 90 alpha family class B member 1 (HSP90AB1), all of which increased their content in response to WD (Fig. 3*C*). This classification in the NIL illustrated how a large number of proteins, including peptides such as OXT, AVP, or PDYN and enzymes such as proprotein convertase subtilisin/kexin type 1 (PCSK1) or A disintegrin and metalloproteinase domain 23 (ADAM23), decrease in their protein content, consistent with secretion from the NIL following WD (Fig. 3*D*).

**Fig. 4:**
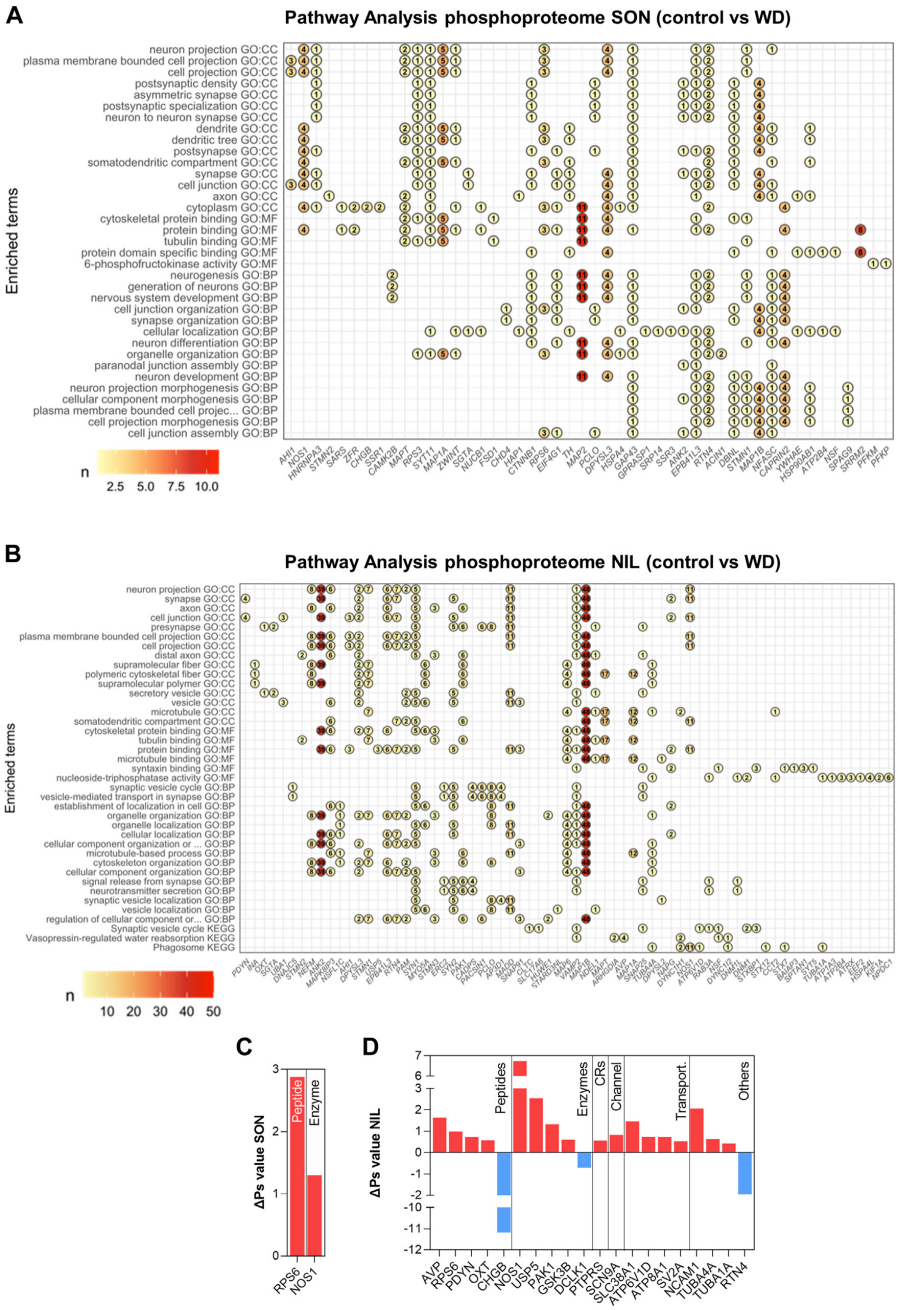
**Pathway analyses and functional classification of the phosphoproteomes of supraoptic nucleus and neurointermediate lobe.***A*, pathway analysis of changes in the SON phosphoproteome as a result of water deprivation (WD) using GO and KEGG databases. Dot plot of up to 15 enriched terms retrieved for each category ranked according to P_Adj_ value from top to bottom in increasing order. The top 10 proteins with most significant phosphorylation changes in a phosphosite are shown as a dot indicating the total number of phosphorylation events in that protein following WD. *B*, pathway analysis of changes in the NIL phosphoproteome as a result of WD using GO and KEGG databases. Dot plot of up to 15 enriched terms retrieved for each category ranked according to P_Adj_ value from top to bottom in increasing order. The top 10 proteins with most significant phosphorylation changes in a phosphosite are shown as a dot indicating the total number of phosphorylation events in that protein following WD. *C*, ΔPs changes in the rat SON as a consequence of WD categorized according to their pharmacological classification or their function as a transcription factor. *D*, ΔPs changes in the rat NIL as a consequence of WD categorized according to their pharmacological classification or their function as a transcription factor. ΔPs, phosphorylation state change; GO, gene ontology; KEGG, Kyoto Encyclopedia of Genes and Genomes; NIL, neurointermediate lobe; SON, supraoptic nucleus.

We next performed pathway analysis using GO and KEGG databases to explore the phosphoproteome changes as a result of WD in the SON and NIL (supplemental Fig. S4). To get insights into the events occurring in MCNs, only differentially phosphorylated proteins with encoding genes among the top 10% most abundant genes in MCNs (supplemental Fig. S1) were included in the analysis. We ranked according to P_Adj_ value up to 15 of the enriched terms identified in each category, and we plotted up to 10 proteins with most significant phosphorylation changes in a phosphosite. In addition, we indicate the total number of phosphorylation events in that protein following WD (Fig. 4). In the SON, this analysis retrieved terms that included “dendrite” and “somatodendritic compartment” in the GO:CC category, “cytoskeletal protein binding” in the GO:MF category, and “synapse organization” in the GO:BP hierarchy (Fig. 4*A* and supplemental Table S3). No significantly enriched terms were identified in the KEGG pathways. In this analysis, we identified phosphorylation changes in several microtubule-associated proteins (MAPs), organizers of the microtubule cytoskeleton (48), including MAP2, MAP1A, MAP1B, and MAPT. We also found phosphorylation events in STMN1, known to control microtubule dynamics (49, 50) and other microtubule-organizing proteins such as drebrin-like (DBNL) (51).

In the NIL, the GO:CC highlighted several terms including “synapse”, “presynapse”, and “secretory vesicle”. The GO:MF category retrieved several terms related to protein binding, and the GO:BP hierarchy highlighted terms related to cellular organization and localization in addition to other terms related to secretion and synaptic signaling and transmission (Fig. 4*B* and supplemental Table S3). Notably, KEGG analysis identified the terms “synaptic vesicle cycle” and “vasopressin-regulated water reabsorption”. As such, phosphorylated proteins were mainly involved in cytoskeleton organization and localization of cellular components including MAP1B, MAP1A, MAPT, and Ankyrin-2 (ANK2) or in secretion and synaptic transmission including vesicle-associated membrane protein 2 (VAMP2), SYN1, and STXBP1. We thus establish that changes in phosphorylation in response to WD in the SON are involved in dendritic cytoskeleton organization, whereas in the NIL, they also regulate synaptic and secretory processes.

We then plotted and classified the proteins with significant changes in ΔPs in response to WD in the SON and the NIL according to their physiological and pharmacological classifications or their identity as transcription factors. In addition to classifying all the proteins with significant ΔPs changes (supplemental Fig. S5), we focused on those encoded by genes in the top 10% of most abundant genes expressed by MCNs. Only S6 and NOS1 ΔPs changed in the SON, while several peptides, enzymes, and transporters underwent changes in phosphorylation in the NIL (Fig. 4, *C* and *D*). The enzyme NOS1 was hyperphosphorylated in both structures (Figs. 4, *C* and *D* and S6). Of note, the hyperphosphorylated peptides AVP, OXT, and PDYN in the NIL also decreased their total protein content in the NIL following WD, suggesting that changes in peptide phosphorylation in the NIL might be related to peptide secretion.

### Phosphorylation Adaptations in the SON and NIL in Response to WD

Pathway analysis of the changes in the phosphoproteome following WD was indicative of important differential adaptations in the SON and the NIL. Phosphorylation modifications in the SON seemed to mediate cytoskeleton remodeling, while in the NIL, they were related to synaptic events. To further explore posttranslational events elicited by WD, we mapped the phosphorylation sites identified by LC-MS/MS to proteins involved in selected ontology terms.

**Fig. 5:**
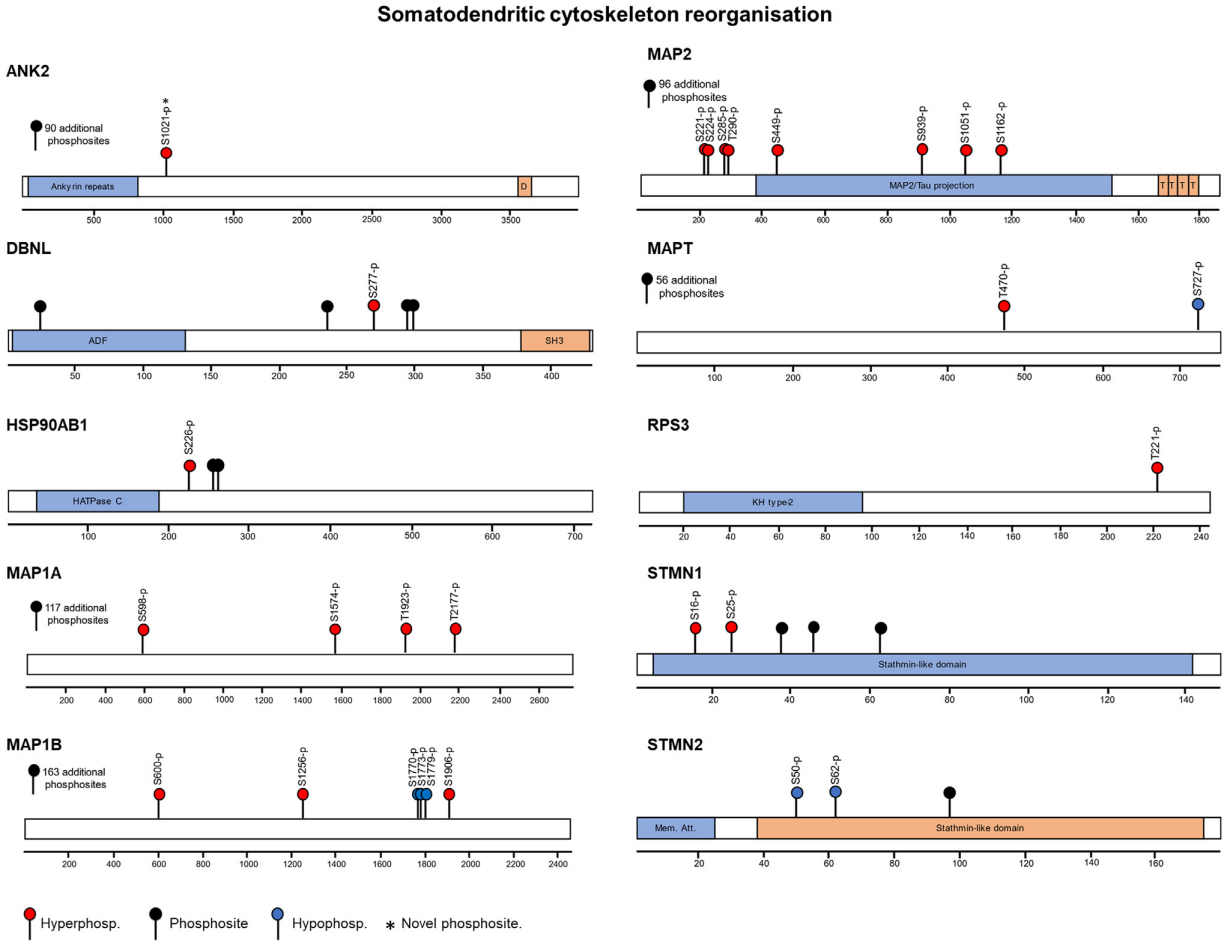
**Phosphorylation events regulating somatodendritic cytoskeleton reorganization in the stimulated supraoptic nucleus.** Mapping the phosphosites and hyper and hypophosphorylation events in response to water deprivation (WD) in proteins involved in microtubule cytoskeleton organization. Protein domains include ADF: actin-depolymerizing factor homology domain; D: Death domain; HATPase C: Histidine kinase-, DNA gyrase B-, and HSP90-like ATPase domain; KH type 2: K Homology type 2 domain; Mem. Att: Membrane attachment domain; SH3, SRC Homology 3 domain; SON, supraoptic nucleus.

In the SON, we explored the common proteins involved in the GO:MF term “cytoskeletal protein binding” and the GO:CC term “somatodendritic compartment” (Fig. 4*A*) to identify those phosphorylation events involved in somatodendritic cytoskeleton remodeling. These included the proteins DBNL, HSP90AB1, MAP1A, MAP1B, MAPT, ribosomal protein S3 (RPS3), and Stathmin 2 (STMN2). We additionally mapped the phosphorylation events in ANK2, MAP2, STMN1, proteins involved in cytoskeletal protein binding. We mapped all phosphosites detected in these proteins in the SON by LC-MS/MS, highlighting those that underwent hyper or hypophosphorylation events (Fig. 5). All these proteins were hyperphosphorylated, with the exception of STMN2 that was hypophosphorylated and MAP1B and MAPT that were both hyper and hypophosphorylated at different residues.

**Fig. 6:**
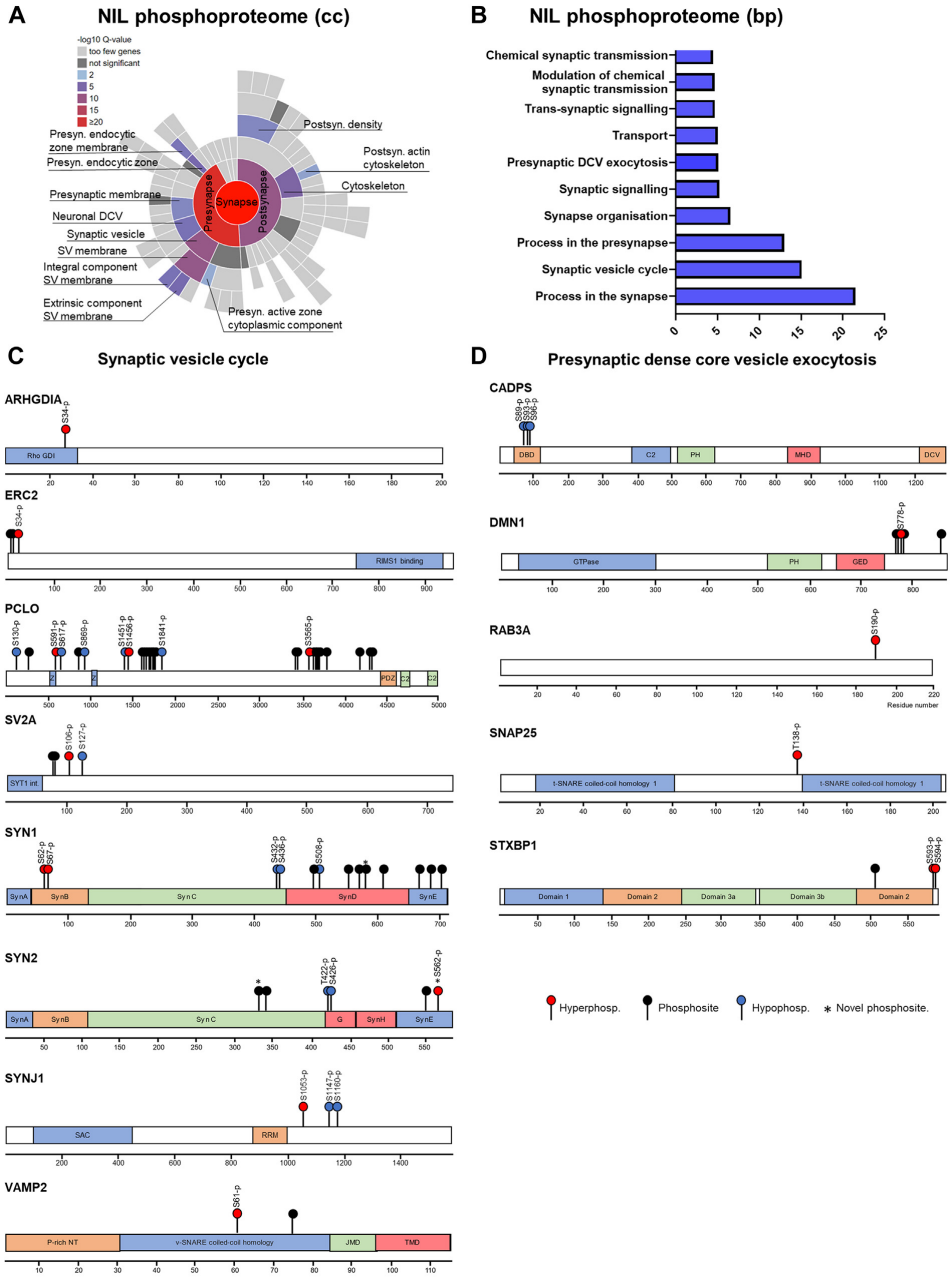
**Phosphorylation events regulating synaptic processes in the stimulated neurointermediate lobe.***A*, SynGO cellular component (cc) enrichment analysis of all the proteins undergoing phosphorylation events in response to water deprivation (WD) in the NIL. *B*, SynGO biological processes (bp) enrichment analysis of all the proteins undergoing phosphorylation events in response to WD in the NIL. *C*, mapping the phosphosites and hyper and hypophosphorylation events in response to WD in proteins involved in the synaptic vesicle cycle. Protein domains include C2: Ca^2+^-dependent C2 domain; G: Pro-rich linker GTPase domain, GTPase domain; JMD: juxta-membrane domain; P-rich NT: proline-rich N-terminal domain; PDZ: post synaptic density domain; Rho GDI: RHO protein GDP dissociation inhibitor; RRM: RRM domain; SAC: SacI homology domain; Syn A, B, C, D, E, F, G, H, J: Synapsin domain A, B, C, D, E, F, G, H, J; SYT1 int: interaction with SYT1 domain; TMD: transmembrane domain; Z: Piccolo Zn-finger. *D*, mapping the phosphosites and hyper and hypophosphorylation events in response to WD in proteins involved in the presynaptic dense core vesicle exocytosis. C2, Ca2+-dependent C2 domain; DBD, dynactin 1 binding domain, DCV, dense core vesicle association domain, GED, Dynamin GTPase effector domain, GTPase, GTPase domain; MHD, Munc13-homology domain; NIL, neurointermediate lobe; PH, Pleckstrin homology domain.

To further explore the phosphorylation events in synapse-related categories and detail synapse-specific changes in the NIL, we ran SynGO analysis (38). For the cellular component ontology, the most enriched terms revealed were at the level of the presynapse (Fig. 6*A*). Biological Process ontology terms highlighted terms such as “process in the presynapse”, “synaptic vesicle cycle”, and “presynaptic DCV exocytosis” (Fig. 6*B* and supplemental Table S4). We mapped all phosphosites detected for some of the proteins involved in the “synaptic vesicle cycle” category which included Rho GDP dissociation inhibitor alpha (ARHGDIA), ERC protein 2 (ERC2), piccolo (PCLO), synaptic vesicle glycoprotein 2A (SV2A), SYN1, SYN2, Synaptojanin-1 (SYNJ1), and VAMP2, among others (Fig. 6*C*). For the term “presynaptic DCV exocytosis”, we mapped the phosphosites for all the proteins involved in this category which included calcium-dependent secretion activator 1 (CADPS), dynamin-1 (DNM1), Ras-related protein Rab3A (RAB3A), synaptosomal-associated protein 25 kDa (SNAP25), and syntaxin-binding protein 1 (STXBP1) (Fig. 6*C*). By mapping the phosphosites to these proteins as well as the hyperphosphorylation and hypophosphorylation sites, we highlight a series of phosphorylation events potentially involved in cytoskeleton organization, the synaptic vesicle cycle, and DCV exocytosis.

### Stimulated SON and NIL Integration

**Fig. 7:**
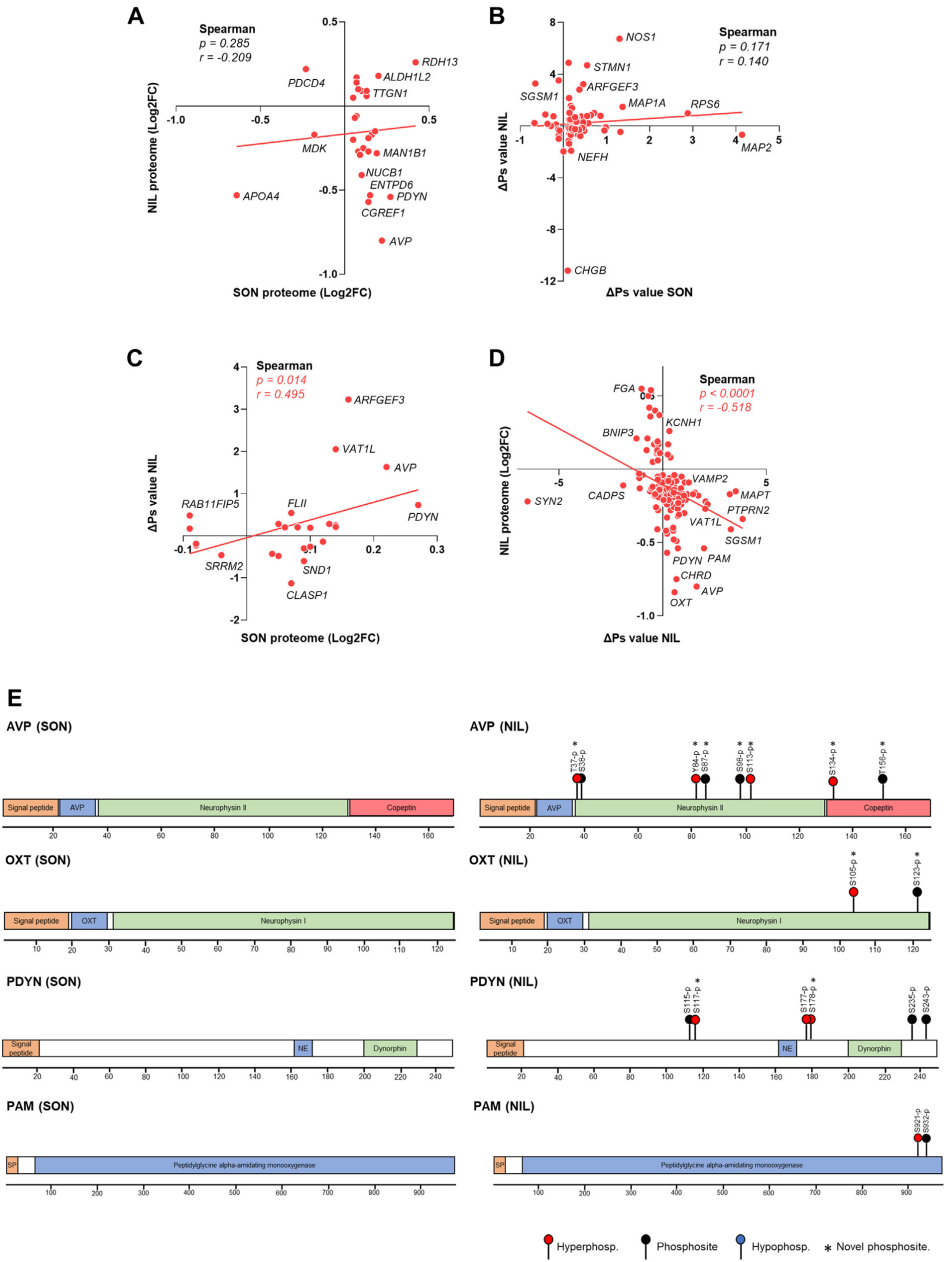
**Multiomic integration of stimulated supraoptic nucleus and neurointermediate lobe.***A*, spearman correlation analysis of Log2FC changes in the rat NIL proteome and SON proteome in response to water deprivation (WD). *B*, spearman correlation analysis of ΔPs changes in the rat NIL and SON in response to WD. *C*, spearman correlation analysis of ΔPs changes in the rat NIL and Log2FC changes in the SON proteome in response to WD. *D*, spearman correlation analysis of Log2FC changes in the rat NIL proteome and ΔPs changes in the NIL in response to WD. *E*, mapping the phosphosites and hyperphosphorylation events in response to WD in Vasopressin-neurophysin 2-copeptin (AVP), Oxytocin-neurophysin 1 (OXT), Proenkephalin-B (PDYN), Peptidylglycine alpha-amidating monooxygenase (PAM) in the SON and NIL. ΔPs, phosphorylation state change; AVP, arginine vasopressin; Log2FC, Log2 fold change; NIL, neurointermediate lobe; SON, supraoptic nucleus.

Next, we performed an integrative analysis of the differential proteomes and phosphoproteomes of the SON and NIL by Spearman correlation analysis. The changes in total proteome between the SON and NIL in response to WD did not correlate (Spearman r = −0.209, *p*-value = 0.285; Fig. 7*A*). A similar pattern was observed with the phosphoproteomes, where the ΔPs between the SON and the NIL did not show a statistically significant correlation (Spearman r = 0.140, *p*-value = 0.171; Fig. 7*B*). This implies cell compartment–specific changes in response to WD. We next explored the relationship between changes in ΔPs in the NIL and changes in the total proteome in response to WD in the SON. Spearman correlation analysis revealed a positive correlation (Spearman r = 0.495, *p*-value = 0.014; Fig. 7*C*). This suggests that, following WD, the SON synthesizes proteins that are transported to the NIL, where they are hyperphosphorylated. In addition, exploring the relationship between changes in ΔPs in the NIL in response to WD and the changes in the total proteome in response to WD in the NIL showed a negative correlation (Spearman r = −0.518, *p*-value = 0.0001; Fig. 7*D*), suggesting that, in response to a stimulus such as WD, hyperphosphorylated proteins are secreted from the NIL into the circulation or are degraded. Among these proteins that undergo hyperphosphorylation and decrease their protein content, consistent with secretion from the NIL, we identified the peptides AVP-neurophysin II, OXT-neurophysin I, PDYN, and the enzyme PAM responsible for peptide C-amidation (46). To further explore the possible role of hyperphosphorylation in the secretion of these proteins, we mapped the identified phosphosites in the SON and the NIL to the protein sequence (Fig. 7*E*). Interestingly, in the SON, none of these proteins underwent any changes in phosphorylation to WD, indeed they presented no phosphorylation events at all. In the NIL, for AVP-neurophysin II, we detected eight phosphosites (seven of them described in the present work from the first time), four of which were hyperphosphorylated in response to WD. Two novel sites were found for OXT-neurophysin I and one of them was hyperphosphorylated in response to WD. For PDYN, we identified six phosphosites, three of which were hyperphosphorylated following stimulus. Of the two phosphosites identified in PAM in the NIL, one was hyperphosphorylated as a consequence of neuronal activation.

### Basal State Transcriptome *Versus* Proteome Integration

**Fig. 8:**
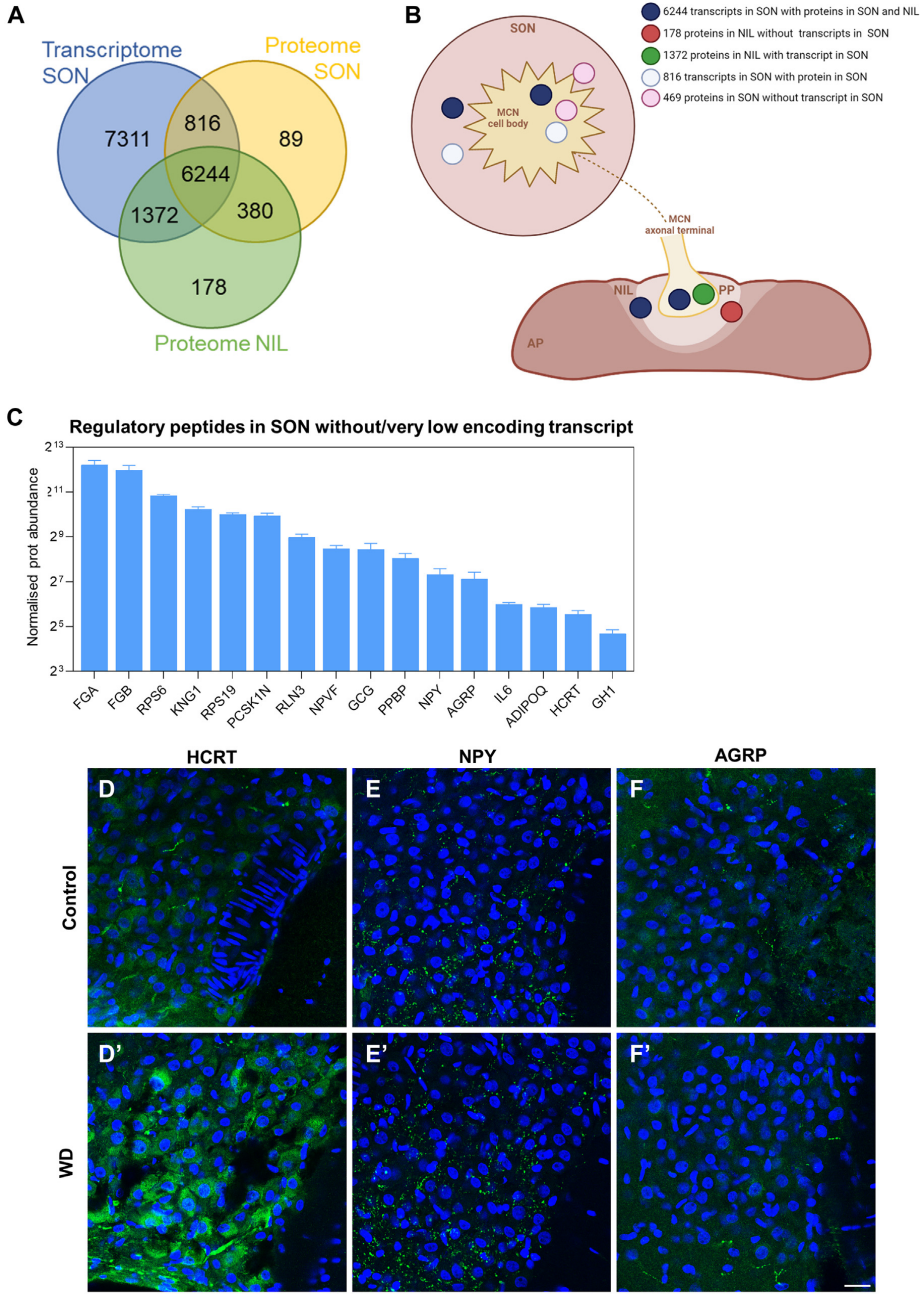
**Basal state transcriptome *versus* proteome integration.***A*, Venn diagram showing the number of overlapping proteins in the supraoptic nucleus (SON) and neurointermediate lobe (NIL) and genes in the SON in control conditions. *B*, schematic representation of the gene and protein dynamics in the SON and NIL in control conditions according to comparisons from the Venn diagram. Generated using BioRender (https://biorender.com/). *C*, regulatory peptides detected in the SON without or very low transcripts in this structure. *D*, *D'*, immunohistochemistry against HCRT in the SON of control and water-deprived (WD) rats. *E, E'*, immunohistochemistry against NPY in the SON of control and WD rats. *F, F'*, immunohistochemistry against AGRP in the SON of control and WD rats. Images are representative of n = 4. Scale bar represents 25 μm. NPY, neuropeptide Y.

We then explored the relationship between the transcriptomes and the proteomes of SONs in the basal condition. In order to generate a complete rat transcriptome, we generated a dataset which included the entire transcriptome determined by RNA-seq of Wistar Han (20) and Sprague Dawley (42) rats in basal conditions (supplemental Fig. S2). We compared transcripts with a mean number of reads >10 with the LC-MS/MS total proteome data from SON and NIL in euhydrated conditions. These comparisons revealed important transcriptome and proteome dynamics in cell bodies and axonal terminals (Fig. 8, *A* and *B*). To begin with, there were 7311 transcripts for which the corresponding encoding proteins were not detected by LC-MS/MS. This can be attributed to the lower sensitivity and dynamic range for detection of proteomics and to technical issues such as protein solubility (52). Interestingly, there were 6244 transcripts in the SON, which encoded proteins detected in both the SON and NIL, likely representing a mix of proteins synthesized in the cell body and transported to the axonal terminals and/or synthesized in different cell populations in the SON and NIL. There were 178 proteins exclusively present in the NIL, without their corresponding transcripts in the SON. With the exception of at least five proteins that may derive from blood contamination [supplemental Fig. S7; (53)], this finding suggests either local synthesis of the remaining proteins in the NIL or inputs from non-SON neurones projecting to the NIL. There were 1372 transcripts in the SON which encoded proteins present in the NIL but not the SON, possibly reflecting protein transport from cell bodies in SON to axonal terminals in NIL. This is further supported by the fact that none of the transcripts encoding these proteins is present in the NIL [supplemental Fig. S7; (54)]. In addition, there were 816 transcripts with proteins present in the SON, but not the NIL, suggesting that these proteins are synthesized in the SON, but are not transported to the PP, instead having unique biological functions in cells bodies and dendrites or that they are produced in SON cells other than MCNs.

Interestingly, there were 469 proteins detected in the SON without a corresponding transcript (Fig. 8, *A* and *B*). We hypothesized these proteins may provide novel insights regarding SON neuronal circuit connectivity, although presence of protein could in some cases be due to contamination from proteins present in the blood. We classified these proteins according to their identity as transcription factors or their physiological or pharmacological categories (supplemental Fig. S5), and we focused on secretory peptides (Fig. 8*C*). We identified hypocretin neuropeptide precursor (HCRT) as a candidate peptide being transported into the SON. Interestingly, the protein abundance of HCRT was increased in response to WD. Immunohistochemistry identified HCRT-positive afferent fibers (Fig. 8*D*), possibly arising from neuronal cell bodies in the lateral and posterior hypothalamus (55) and was indicative of increased HCRT protein content in the SON (Fig. 8*D*, D’). In addition, neuropeptide Y (NPY), agouti-related protein (AGRP), and preproglucagon (GCG) were detected at the protein but not mRNA level. The presence of glucagon-like peptide 1 (GLP1) in afferent fibers within the SON is well known (56), possibly arising from the nucleus tractus solitarius (57). Immunostaining of NPY revealed axonal terminals containing NPY peptide in the SON (Fig. 8*E*), possibly projecting from other hypothalamic structures (58, 59). In agreement with LC-MS/MS, no changes in NPY immunolabeling were observed in response to WD (Fig. 8*E*, E’). We also detected the presence of ARGP-containing axonal fibers in the SON (Fig. 8*F*) and again observed no change in response to WD (Fig. 8*F*, F’). Thus, we have identified several signaling proteins that could potentially be transported into the SON from other brain regions to mediate functional outcomes.

## Discussion

In this work, we have explored the proteomes and phosphoproteomes of different neuronal compartments in the SON and NIL under basal and stimulated conditions. Through comparisons with corresponding SON transcriptomes, we bring a novel perspective to transcriptome, proteome, and phosphoproteome dynamics in this uniquely tractable model neuronal system.

While it is well known that proteins synthesized in SON cell bodies are transported to axonal terminals in the PP, our data globally quantifies this phenomenon. When stimulated, MCNs change their steady-state RNA levels and proteomes. In response to WD, proteome and phosphoproteome dynamics differ between the SON and NIL, showing that each neuronal compartment adapts in a very distinctive way to facilitate changes to cell secretory requirements. In the SON, this involves the synthesis of new proteins to meet the demand for newly synthesized peptides (such as AVP and OXT) and associated secretory machinery. The protein phosphorylations in the SON in WD seem to mediate separate functions related to cytoskeleton organization. In particular, MAP1B S1256 phosphorylation site has already been shown to contribute to the regulation of microtubule dynamics (60). Also, phosphorylation of the STMN1 phosphosites identified in this work has been shown to promote microtubule stability (61), and S25-p and S38-p have been found to be phosphorylated in response to hyperosmotic stress (62). It has been demonstrated that MCNs have a distinctive cytoskeleton composed of a layer of actin filaments beneath the plasma membrane and a unique network of cytoplasmic actin filaments and microtubule interweaved scaffold (63, 64). The cytoskeleton of MCNs undergoes reorganization in response to hyperosmotic stimuli, which is believed to underlie the intrinsic osmosensitivity of MCNs (63, 64, 65). Our data suggests that phosphorylation events in response to WD in the SON may contribute to changes in the cytoskeletal organization in MCNs. Furthermore, we have also mapped the phosphosites that change in response to WD in proteins involved in cytoskeleton remodeling, such as MAP and other microtubule-organizing proteins, providing a global overview of the phosphorylation events mediating cytoskeleton reorganization.

In the NIL, pathway analysis for changes in the proteome and phosphoproteome in WD informed of adaptations to the synaptic vesicle cycle instrumental for secretion. By mapping phosphosites and responses to stimuli, we provide a global overview of the phosphorylation events involved in the synaptic vesicle cycle and secretion. We report a catalog of the phosphorylation events that take place in known proteins of the synaptic vesicle cycle, synaptic vesicle clustering, and synaptic exocytosis. These include critical proteins of the synaptic vesicle cycle such as PCLO and proteins from the VAMP, synaptotagmin, and synapsin families. Although most of the phosphorylation events that we describe have no currently known functions, some have already been explored. Phosphorylation of SYN1 at S62/67 causes the dissociation of synaptic vesicles from the actin cytoskeleton resulting on their mobilization from the reserve pool to the release-ready pool (66). Phosphorylation of SNAP25 at T138 inhibits assembly of the SNARE complex and exocytosis (67) but increases the size of releasable vesicle pools (68). SYNJ1 is dephosphorylated at S1160 upon neuronal depolarization to stimulate the inositol 5-phosphatase activity of SYNJ1 and control the efficiency of the depolarization-exocytosis coupling of synaptic vesicles (69). These findings further support the role of these phosphorylation changes in the synaptic vesicle cycle in the HNS. We also provide a catalog of the phosphorylation events taking place for proteins involved in “presynaptic DCV exocytosis”, which include the proteins CADPS, required for Ca^2+^-activated DCV exocytosis (70), DNM1, RAB3A, and the syntaxin-binding protein STXBP1. It has been described that DNM1 is constitutively phosphorylated at S778. This residue is dephosphorylated following neuronal stimulus to facilitate synaptic vesicle endocytosis, which is necessary to maintain a pool of synaptic vesicles within nerve terminals after exocytosis, following which it is rephosphorylated to allow for the next round of synaptic vesicle endocytosis (71).

The negative correlation between the ΔPs and the proteome in the NIL as a result of WD suggests that hyperphosphorylation of proteins maybe a key component for neuropeptide processing and/or secretion from nerve terminals. In particular, in the AVP and PDYN precursor proteins, we have identified phosphorylation events next to the cleavage sites suggesting that changes in phosphorylation might regulate processing, as it has already been observed for gastrin (72).

The protein NOS1 had high ΔP values both in the SON and the NIL. Interestingly, by mapping the phosphorylation sites and their changes to WD, both in the SON and NIL, we show cell compartment–specific phosphorylation patterns. We detected hyperphosphorylation events in the flavin mononucleotide–binding domain in the SON and NIL as a result of WD, where increased phosphorylation at S847 in the NIL, but not the SON, has been shown to reduce NOS1 activity by inhibiting the binding of Ca^2+^ to the calmodulin domain (73, 74). In addition, NOS1 phosphorylation at S1412 in the NADPH-binding domain (hyperphosphorylated in SON, but not NIL) has been shown to increase the activity of NOS1 (75, 76). It has been suggested that hyperosmotic stimulation induces NO production in MCNs in the SON (77, 78), reducing AVP and OXT secretion as a feedback compensatory mechanism to prevent oversecretion of these peptides (79). The different phosphorylation events in NOS1 between the NIL and SON identified in this study and the implications regarding the differential activities of this enzyme in discrete cellular structures can contribute to fully understand the role of NOS1 in MCNs and other neuronal systems.

In short, in response to WD, peptide synthesis (*i.e.* AVP and PDYN) is increased in the SON, and peptides are released from the NIL (observed as a decrease in protein content; *i.e.* AVP, OXT, PDYN). In order to mediate this, the SON responds by producing proteins that facilitate the synthesis of neuropeptides and their processing. These neuropeptides are transported to the axonal terminals where they are hyperphosphorylated and released, due to phosphorylation adaptations in the NIL. The data illustrates that studying the transcriptome responses of a cell type to understand its function gives limited information and that the proteome and phosphoproteome responses should also be explored. In addition, it illustrates that in complex cellular types, such as neurones, these responses need to be considered separately between the cell body and axonal terminals, as they are indeed very different.

We have also identified a number of proteins in the SON without the presence of their corresponding transcripts, and validated proteins known to be found in afferents. The SON expresses the HCRT receptors *Hcrtr1* and *Hcrtr2*, the NPY receptors *Npy1r*, *Npy2r* and *Npy5r*, and the AGRP receptors *Mc3r* and *Mc4r*, the latter even increases in response to WD (20), in agreement with regulation by these neuropeptides. The increase in HCRT in WD supports a role for this neuropeptide circuit in the control on MCN functions. Interestingly, HCRT regulates the sleep-wake cycle (80), and stimulation of AVP neurones in the PVN induces wakefulness *via* lateral hypothalamic orexin neurones. It has been demonstrated that WD reduces motor activity and increases slow-wave sleep (81), so HCRT could potentially be mediating these effects.

In order to better understand the biological functions of neurones, a comprehensive multiomic understanding of activity-dependent neuronal cellular pathways and processes in distinct cellular and subcellular compartments is needed. But in mammals, this is easier said than done. We have taken advantage of the unique anatomical organization of the HNS to document transcriptome, proteome, and phosphoproteome dynamics in this structure in response to neuronal activation. These data show how different compartments of the HNS respond to stimulation. This multiomic approach provides a wealth of new knowledge about how neuronal stimulation reshapes the proteome and phosphoproteome to be utilized by the neuroscience community and beyond.

## Data Availability

All data are available in the manuscript or supplemental information. The LC-MS/MS proteomics and phosphoproteomics data have been deposited to the ProteomeXchange Consortium *via* the PRIDE (33) partner repository with the dataset identifier PXD033401. Any additional information is available from the lead contact upon request.

## Supplemental data

This article contains supplemental data (20, 34, 42, 53, 54).

## Conflict of interest

The authors declare that they have no conflicts of interest.

## Supplemental Data

Supplemental figures

Supplemental table captions
