# Supplementary material for: Translational and Posttranslational Dynamics in a Model Peptidergic System
Source: Mol Cell Proteomics. 2023 Apr 6;22(5):100544. doi: 10.1016/j.mcpro.2023.100544 (PMC10205546; doi:10.1016/j.mcpro.2023.100544)
Supplement: Supplemental figures [file mmc5.pdf]

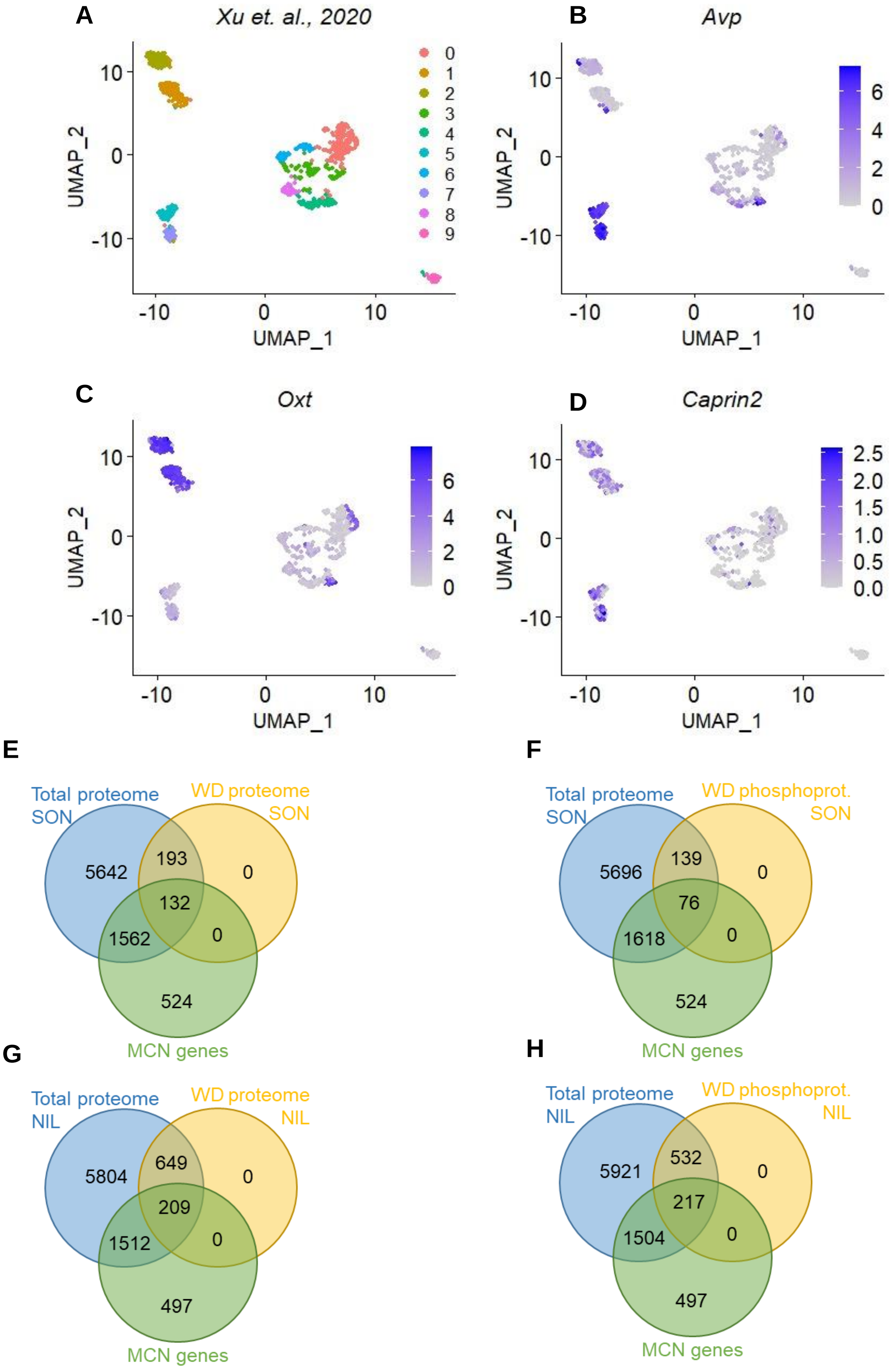

Figure S1. Single-cell reanalysis of the mouse PVN from Xu et al., 2020.
(A) Uniform Manifold Approximation and Projection (UMAP) plot of all 706 neurons used for Xu et al., 2020 analysis. Our clustering analysis revealed ten neuronal type identities.
(B) UMAP plot showing the high expression of Avp on clusters 5 and 7.
(C) UMAP plot showing the high expression of Oxt on clusters 1 and 2.
(D) UMAP plot showing the high expression of Caprin2 (a marker for the magnocellular neurons (MCN)– Baréz-López et al., 2022) on clusters 1, 2, 5 and 7.
(E) Venn diagram illustrating common elements between the top 10% genes expressed in MCN, the genes encoding the total SON proteome, the genes encoding the proteins that undergo changes in their content in response to water deprivation (WD) in the SON.
(F) Venn diagram illustrating common elements between the top 10% genes expressed in MCN, the genes encoding the total SON proteome, the genes encoding the proteins that undergo changes in phosphorylation in response to WD in the SON.
(G) Venn diagram illustrating common elements between the top 10% genes expressed in MCN, the genes encoding the total NIL proteome, the genes encoding the proteins that undergo changes in their content in response to WD in the NIL.
(H) Venn diagram illustrating common elements between the top 10% genes expressed in MCN, the genes encoding the total NIL proteome, the genes encoding the proteins that undergo changes in phosphorylation in response to WD in the NIL.

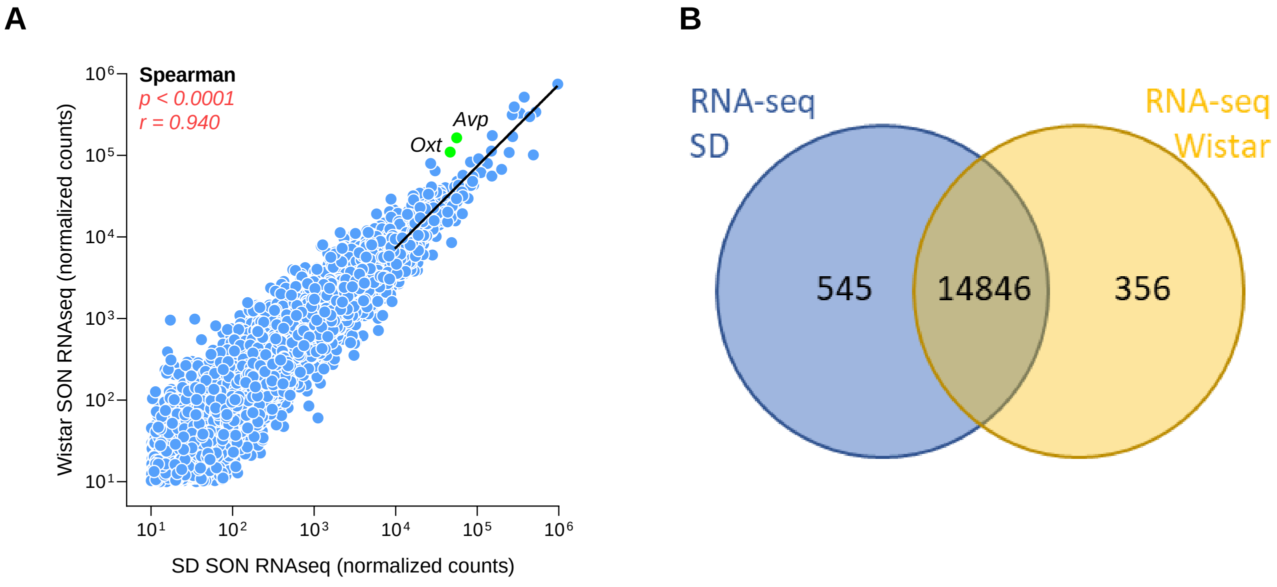

Figure S2. Comparison of SON transcriptome from Wistar Han (Pauza et al., 2021) and Sprague Dawley rats (Bárez-López et al., 2022).

A) Spearman correlation analyses show the similar expression level of the commonly expressed genes in the SON of these two rat strains.

B) Venn diagram showing the highly conserved transcriptome between Wistar Han and Sprague Dawley rats.

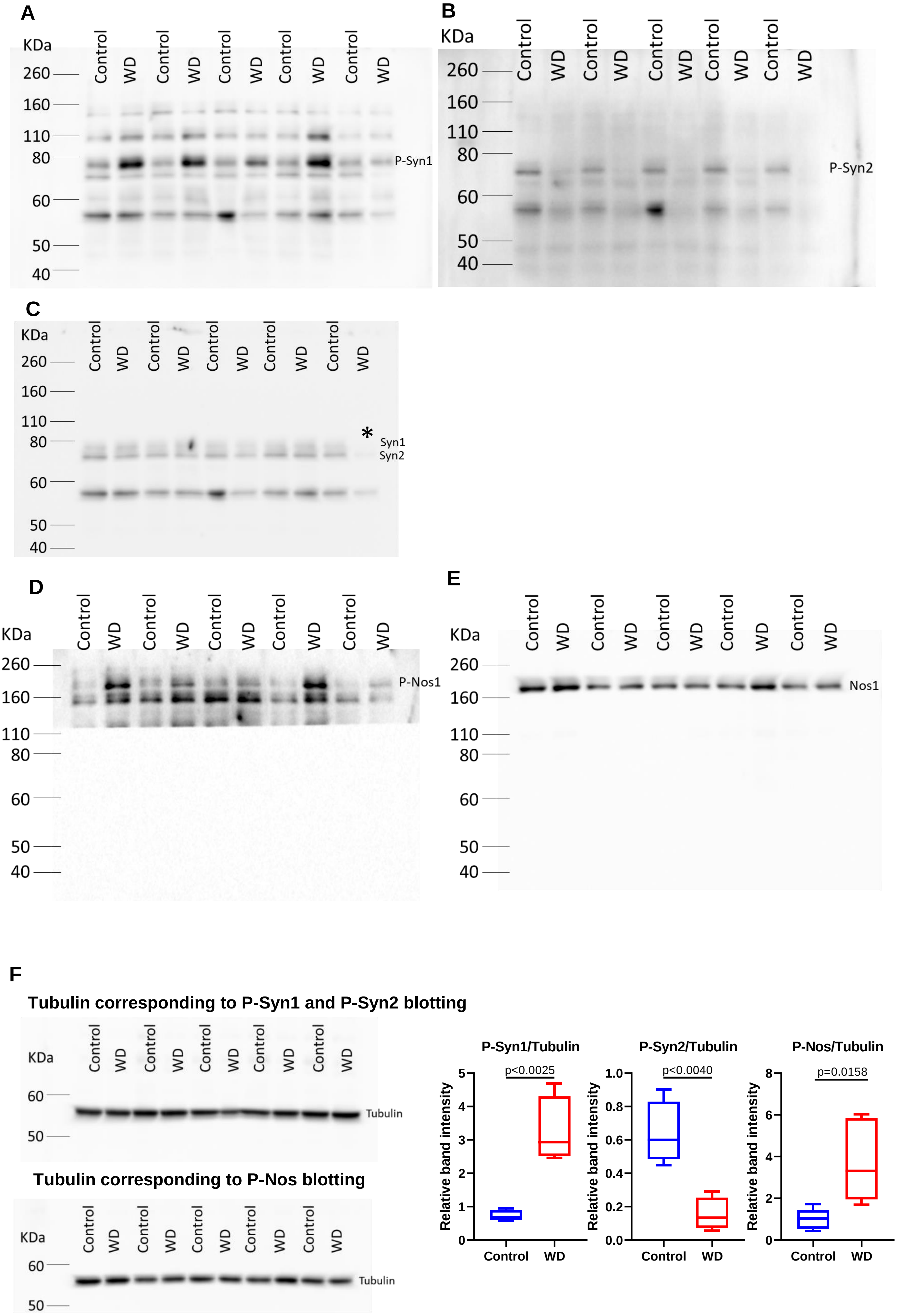

Figure S3. Complete Western Blots with control and 48 hours water deprived (WD) blotted against P-Syn1 (A), P-Syn2 (B), Syn (C), P-Nos (D) and Nos (E).

(C) Due to the lack of Syn signal in the last lane (*), this sample was excluded from the P-Syn1 and P-Syn2 analysis.

(D) This membrane was covered during signal detection to prevent signal bleed-through from previous rounds of protein detection.

(F) Normalising the signal to tubulin rendered the same results as normalising to total Syn and total Nos.

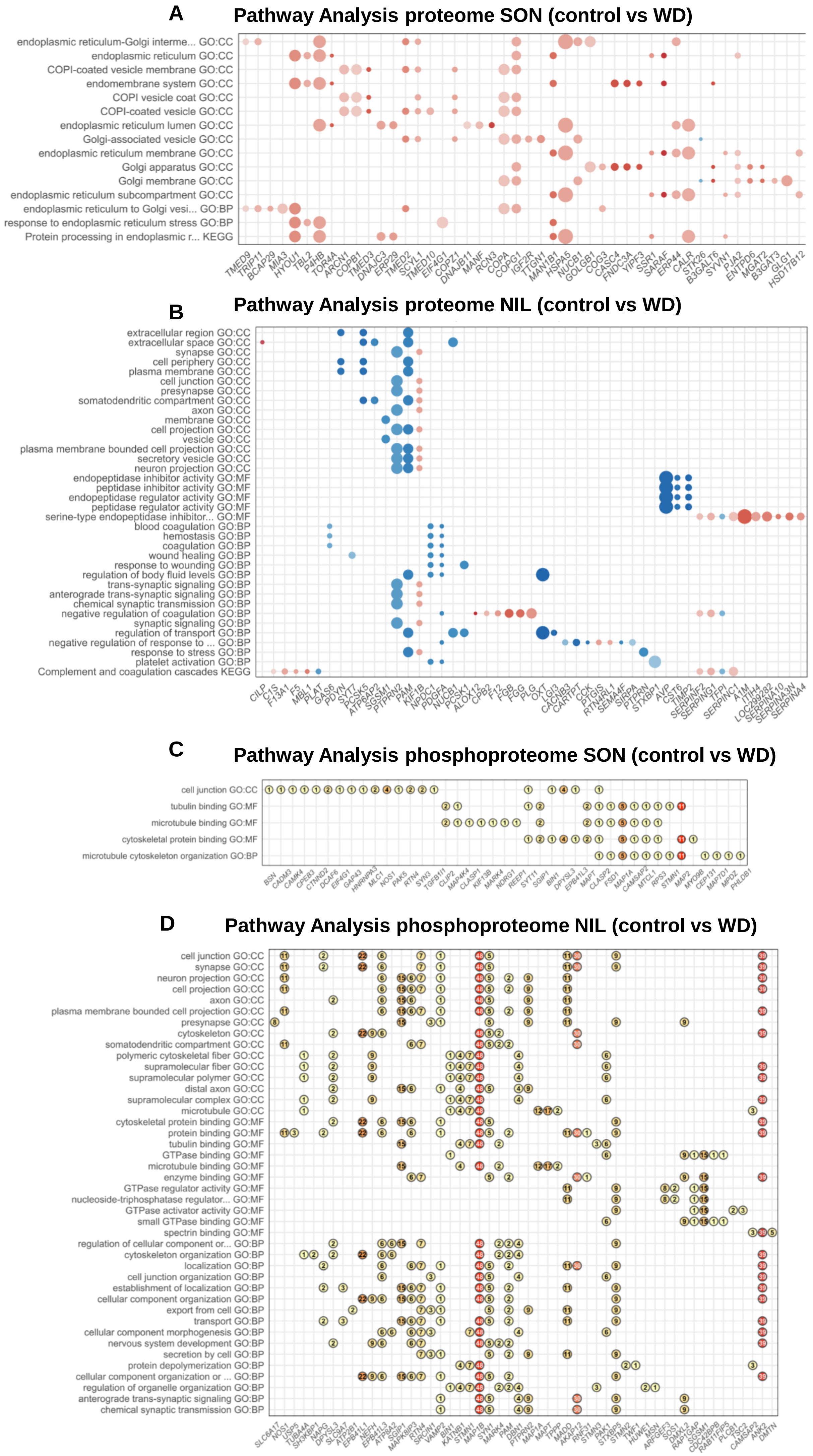

Figure S4. Pathway analyses of the proteomes and phosphoproteomes of supraoptic nucleus (SON) and neurointermediate lobe (NIL).

(A) Pathway analysis of changes in the SON proteome as a result of water deprivation (WD) using GO and KEGG databases.

(B) Pathway analysis of changes in the NIL proteome as a result of WD using GO and KEGG databases. Further tests revealed that synaptic terms are related to down-regulated proteins whereas coagulation and peptidase regulation terms are related to up-regulated proteins in response to water deprivation (WD) .

(C) Pathway analysis of changes in the SON phosphoproteome as a result of WD using GO and KEGG databases.

(D) Pathway analysis of changes in the NIL phosphoproteome as a result of WD using GO and KEGG databases.

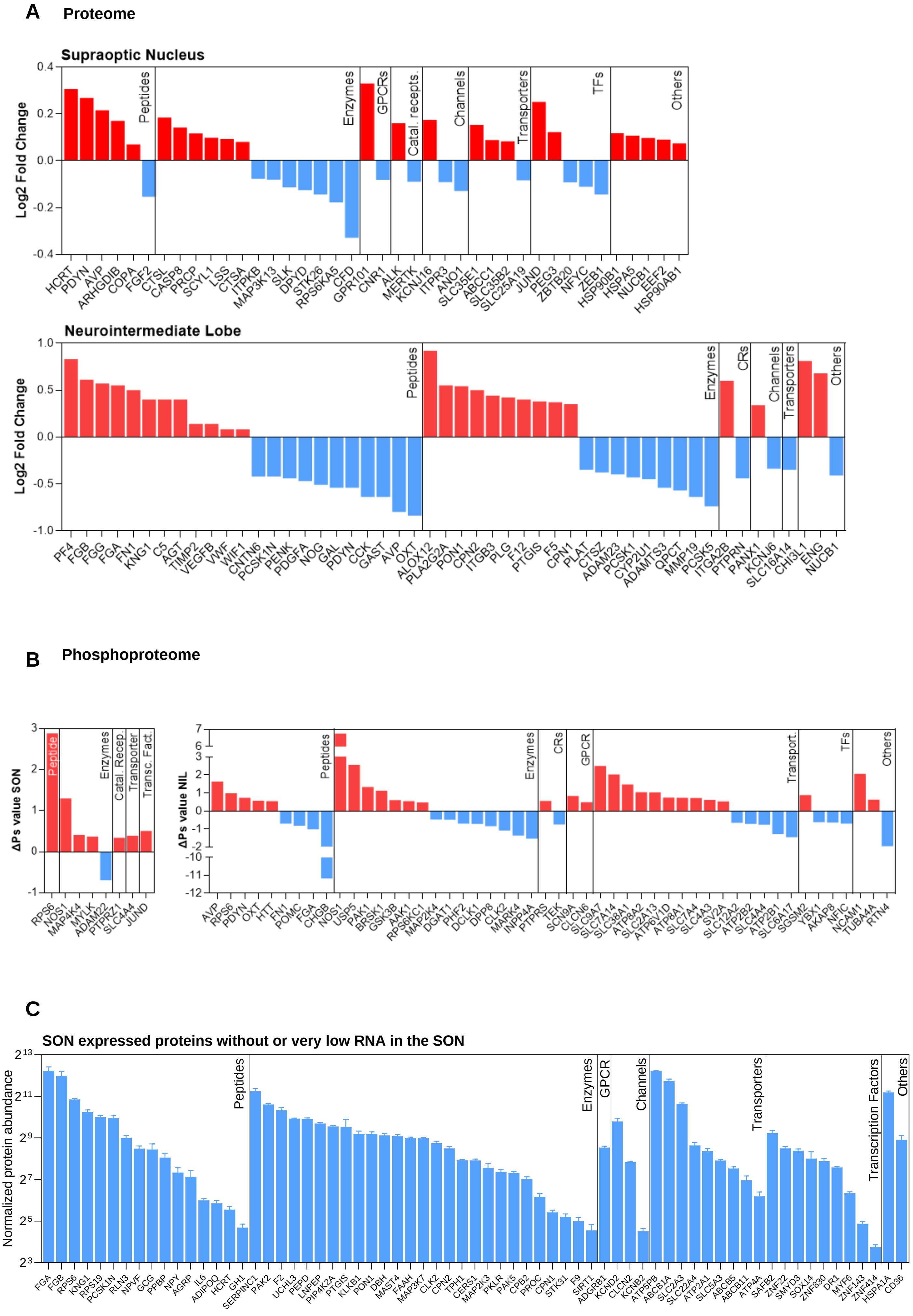

Figure S5. S4 Classification of proteins according to their pharmacological category or their function as a transcription factors for (A) all proteome Log2FC changes as a consequence of water deprivation (WD) in the rat supraoptic nucleus (SON) and neurointermediate lobe (NIL), (B) global overall phosphorylation state changes (ΔPs) as a consequence of WD in the rat SON and NIL, (C) all proteins detected in the SON without or very low transcripts in this structure.

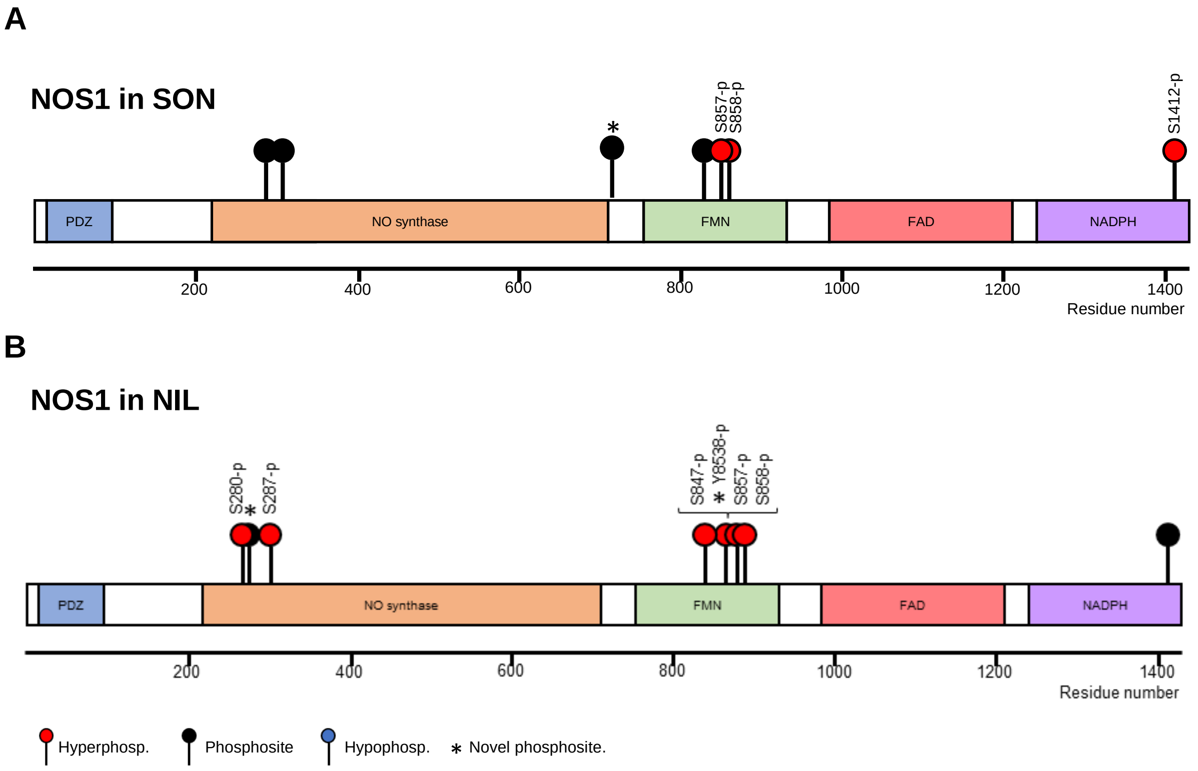

Figure S6. Mapping the phosphosites and hyper and hypophosphorylation events in NOS1 in response to water deprivation in the (A) SON and (B) NIL.

A

B

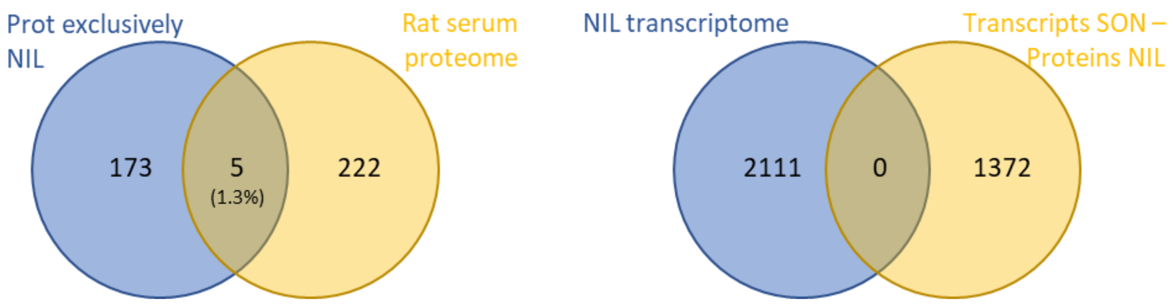

Figure S7. (A) Venn diagram showing overlap between proteins present exclusively in neurointermediate lobe (NIL) without their corresponding transcript in the supraoptic nucleus (SON), with rat serum proteome identified by Ma et al., (2018). **(B)** Venn diagram showing no overlap between the NIL transcriptome identified by single Cell RNA-seq by Chen et al., (2020) and those proteins present in the NIL (but not the SON) which encoding transcripts are detected in the SON.
