# Supplementary material for: Translational and Posttranslational Dynamics in a Model Peptidergic System
Source: Mol Cell Proteomics. 2023 Apr 6;22(5):100544. doi: 10.1016/j.mcpro.2023.100544 (PMC10205546; doi:10.1016/j.mcpro.2023.100544)
Supplement: Supplemental table captions [file mmc6.docx]

**Translational and post-translational dynamics in a model peptidergic system**

Soledad Bárez-López, André S. Mecawi, Natasha Bryan, Audrys G. Pauža, Victor J. Duque, Benjamin T. Gillard, David Murphy and Michael P. Greenwood

**Supplemental table captions**

**Supplemental table S1**: Nano-LC Mass Spectrometry output listing all proteins detected in the supraoptic nucleus (SON) as well as differentially produced proteins and phosphosites between control and water deprived rats in the SON.

**Supplemental table S2**: Nano-LC Mass Spectrometry output listing all proteins detected in the neurointermediate lobe (NIL) as well as differentially produced proteins and phosphosites between control and water deprived rats in the NIL.

**Supplemental table S3**: Pathway analysis output of the differential produced proteins and differentially phosphorylated proteins between control and water deprived supraoptic (SON) and neurointermediate lobe (NIL) samples by interrogating GO and KEGG databases based on the top 10% of abundant protein encoding genes expressed in MCNs. Databases searched included GO:Cellular Component (GO:CC), GO:Molecular Function (GO:MF) and GO:Biological Process (GO:BP) and Kyoto Encyclopedia of Genes and Genomes (KEGG).

**Supplemental table S4**: Pathway analysis output of the differential differentially phosphorylated proteins between control and water deprived neurointermediate lobe (NIL) samples by interrogating SynGO database. Cellular Component (CC), Biological Process (BP)
